# A positive feedback loop between TKT and c-Myc drives TACE resistance in hepatocellular carcinoma

**DOI:** 10.1038/s41420-026-03125-8

**Published:** 2026-04-21

**Authors:** Yifu Xiao, Mingyu Liu, Ying Zhou, Yunyuan Bao, Guoqing Zhang, Banglong Xu, Wenjie Zheng, Hui Zhao

**Affiliations:** 1 Department of Interventional Radiology, Affiliated Hospital of Nantong University, Medical School of Nantong University, Nantong, China; 2 Research Center of Clinical Medicine, Affiliated Hospital of Nantong University, Medical School of Nantong University, Nantong, China

**Keywords:** Cancer metabolism, Ubiquitylation

## Abstract

Hepatocellular carcinoma (HCC) often receives transarterial chemoembolization (TACE), yet clinical benefit is limited by resistance driven by ischemia-induced adaptations. This study explains how transketolase (TKT) in the pentose phosphate pathway (PPP) modulates HCC progression and TACE refractoriness, and clarifies its mechanistic connection to oncogenic signaling. We integrated transcriptomic screening with analyses of TACE patient specimens, and performed gain and loss of function experiments across HCC cell lines. Functional assays, RNA-seq with pathway enrichment, western blotting, immunofluorescence, cycloheximide-chase and ubiquitination assays, and an orthotopic VX2 rabbit TACE model with imaging and immunohistochemistry were used. TKT emerged as a hub gene, was elevated in TACE-resistant patients, and promoted proliferation, migration, invasion, epithelial–mesenchymal transition, and apoptosis resistance. Mechanistically, TKT associates with RAF1, promoting phosphorylation of c-Raf at Ser338 and subsequent ERK activation, and stabilizes c-Myc by enhancing Ser62 phosphorylation and reducing ubiquitin-mediated degradation. Additionally, c-Myc enhances the transcriptional expression of TKT, creating a positive feedback loop between TKT and c-Myc. These findings identify a TKT/c-Myc positive feedback loop that underlies TACE resistance and HCC progression, nominating TKT as a biomarker of refractoriness and a therapeutic target to improve locoregional treatment outcomes.

## Introduction

Liver cancer ranks as the sixth most common malignancy globally and the third leading cause of cancer-related deaths [1, 2]. Among its subtypes, HCC constitutes ~80% of all primary liver cancer cases. China contributes nearly half of the global incidence and mortality burden [3, 4]. Although curative surgical resection is feasible in patients diagnosed at an early stage, the overall five-year survival rate remains ~12.1%, highlighting the disease’s poor prognosis [5]. For intermediate-stage HCC, as classified by the Barcelona Clinic Liver Cancer stage B(BCLC-B), TACE is considered the standard locoregional therapy, and serves as a pivotal modality in both bridging to transplantation and implementing downstaging strategies [4]. Given the high risk of postoperative recurrence, both adjuvant TACE following resection and repeat TACE after recurrence are frequently adopted in clinical practice, though their survival benefits remain heterogeneous and controversial [6]. Despite achieving an objective response rate of ~50–60%, about 40% of patients either fail to respond or experience rapid recurrence, leading to substantial variability in treatment outcomes [7]. The acute ischemia and hypoxia induced by TACE can activate the HIF-1/2 signaling axis, which promotes angiogenesis, immunosuppression, epithelial–mesenchymal transition, and metabolic reprogramming, thereby driving resistance to TACE [8].

Metabolic adaptations are a hallmark of malignant cells [9], with the PPP supplying nucleotide precursors and NADPH to sustain biosynthesis and redox homeostasis under stress [10]. Within this pathway, TKT is a key enzyme in the non-oxidative branch that acts as a rate-limiting factor [11] and is frequently elevated in HCC, where it is associated with unfavorable outcomes [12]. In addition to its catalytic activity, nuclear-localized TKT aids tumor development by engaging transcriptional regulators and kinases, and its presence in the nucleus is associated with a worse prognosis in HCC [13]. In parallel, c-Myc abundance is tightly regulated post-translationally: ERK-mediated phosphorylation at Ser62 stabilizes c-Myc, whereas sequential phosphorylation at Thr58 by GSK3β primes it for ubiquitin–proteasome-mediated degradation [14, 15]. Inhibition of the MEK/ERK pathway destabilizes c-Myc, thereby reducing proliferation and anchorage-independent growth in tumor cells [16]. Collectively, these findings indicate that PPP/TKT activity and c-Myc proteostasis converge critically in HCC pathobiology; however, the mechanisms by which TKT influences oncogenic transcriptional regulation remain incompletely defined.

The goal of this study is to systematically explore how TKT influences HCC, its relationship with TACE response, and the molecular processes behind the TKT–c-Myc positive feedback loop contributing to TACE resistance. Our findings demonstrate that TKT promotes HCC progression by enhancing ERK-mediated stabilization of c-Myc. In turn, c-Myc accumulation transcriptionally reactivates TKT, forming a positive feedback circuit that exacerbates HCC progression. Targeting this pathway may offer a potential strategy for combining TACE with metabolic treatments.

## Results

### TKT is highly expressed in TACE-resistant HCC patients

**Fig. 1:**
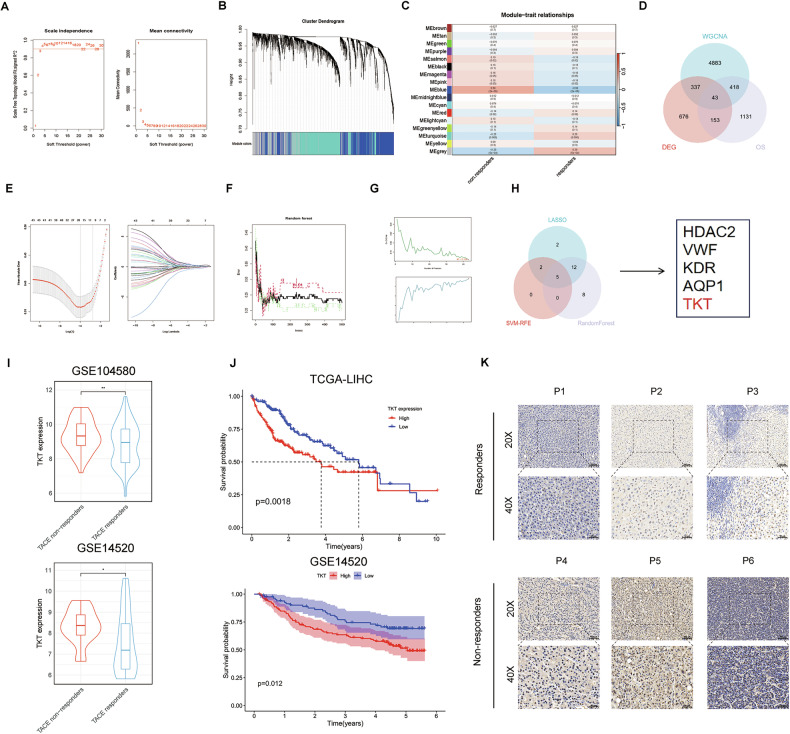
Identification of TKT as a hub gene linked to TACE resistance in HCC. **A** Scale-free topology model fit for WGCNA. **B** Gene dendrogram with module colors. **C** Heatmap of correlations between module eigengenes and TACE response. **D** Venn diagram of WGCNA modules, DEGs, and oxidative stress-related genes. **E**–**G** LASSO, random forest, and SVM-RFE analyses identifying candidate hub genes. **H** Intersection of machine-learning algorithms yielding five hub gene). **I** Boxplots of TKT expression in responders vs. non-responders. **J** Kaplan–Meier analysis of overall survival by TKT expression. **K** Representative IHC of TKT in tumors from responders and non-responders.

By applying weighted gene co-expression network analysis (WGCNA), 17 distinct gene modules were identified with the dynamic tree-cutting method, each assigned a specific color (Fig. 1A–C, Supplementary Fig. S1A). Among them, the blue module showed the most significant correlation, containing a total of 5681 genes (Supplementary Fig. S1B). Differential expression analysis further identified 1209 genes (Supplementary Fig. S1C, D), and intersection with the oxidative stress-related gene set (OS) yielded 43 overlapping genes (Fig. 1D). Through the application of three machine learning algorithms, five hub genes were screened out (Fig. 1E–H, Supplementary Fig. S1E). Considering previous findings that TACE resistance is closely associated with hypoxia-induced metabolic reprogramming, TKT was selected as the candidate gene for further investigation in HCC TACE resistance. Notably, patients with TACE resistance exhibited higher TKT expression (Fig. 1I). Kaplan–Meier analysis showed that elevated TKT was associated with poorer overall survival (Fig. 1J). Consistently, IHC of specimens from TACE-treated patients revealed increased TKT levels in resistant tumors (Fig. 1K), supporting TKT as a prognostic biomarker.

### TKT enhances the tumorigenic capacity of HCC cells in vitro

**Fig. 2:**
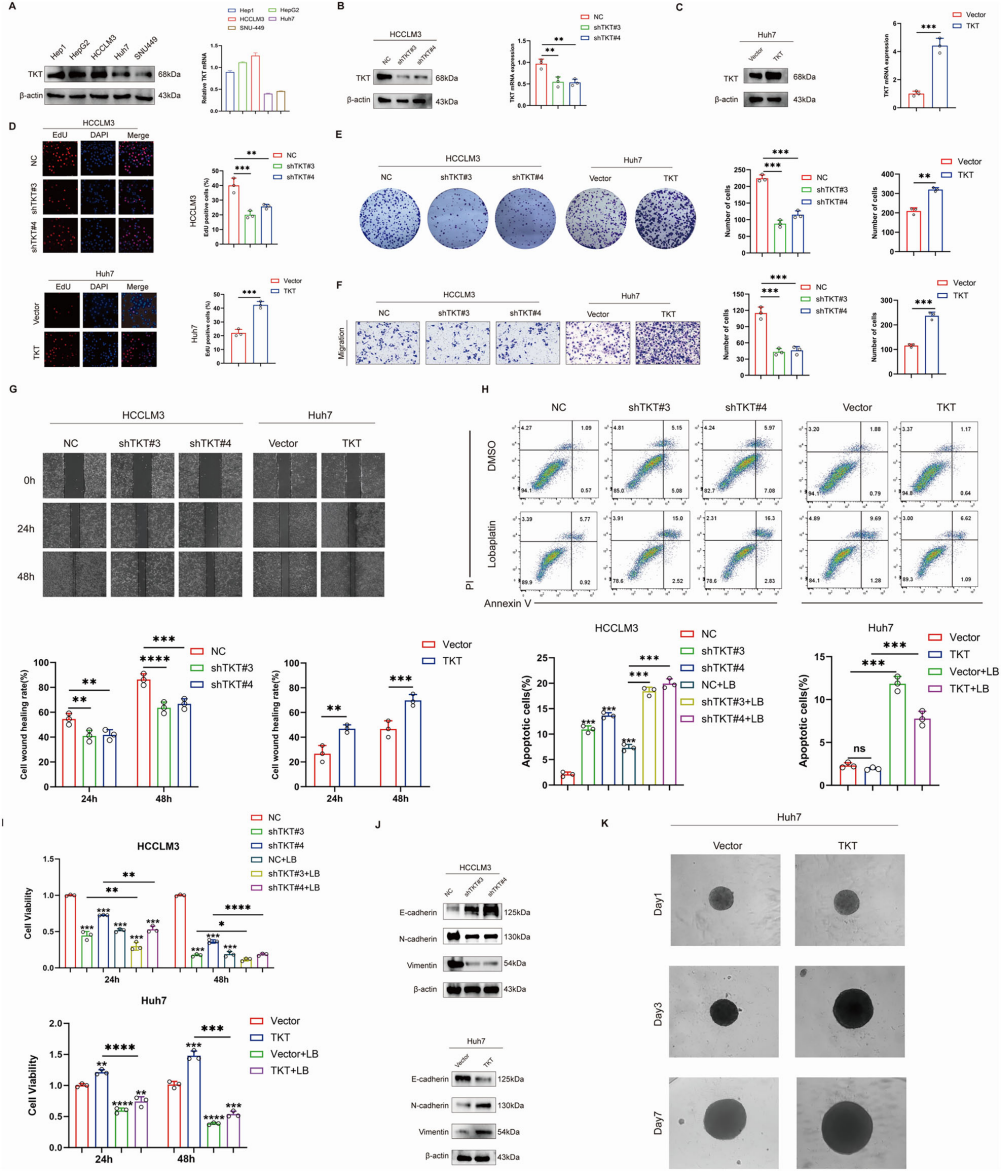
TKT promotes proliferation, migration, invasion, and apoptosis resistance in HCC cells. **A**–**C** Western blot of TKT expression and validation of knockdown/overexpression. **D**, **E** EdU and colony formation assays showing TKT enhances proliferation. **F**, **G** Transwell and wound healing assays showing TKT promotes migration. **H**, **I** Apoptosis and CCK-8 assays under lobaplatin treatment showing TKT confers survival. **J** Western blot of EMT markers. **K** Overexpression of TKT increased the ability of Huh7 cells to form tumoursphere structures.

mRNA and Protein expression of TKT across five HCC cell lines were examined by qRT-PCR and Western blotting. Results revealed significantly elevated expression of TKT in HCCLM3 cells and low expression in Huh7 cells (Fig. 2A). To elucidate its functional role, TKT was silenced in HCCLM3 cells with high endogenous expression, while it was overexpressed in Huh7 cells with low basal expression. Both Western blotting and RT-qPCR confirmed effective knockdown and overexpression (Fig. 2B, C). Functional assays demonstrated that TKT promoted cell proliferation, migration, invasion, and resistance to apoptosis. EdU staining showed that TKT overexpression markedly enhanced proliferation in Huh7 cells, whereas TKT knockdown significantly suppressed proliferation in HCCLM3 cells (Fig. 2D). Consistently, colony formation assays revealed a greater number of colonies in the TKT-overexpressing Huh7 group and a significant reduction in colonies in TKT-silenced HCCLM3 cells (Fig. 2E). Migration and invasion assays further supported these findings. Both Transwell and wound healing assays indicated that TKT overexpression increased the migratory potential of Huh7 cells, whereas TKT knockdown impaired migration in HCCLM3 cells (Fig. 2F, G). Western blotting confirmed that TKT promoted EMT, as evidenced by decreased E-cadherin and increased N-cadherin and Vimentin expression (Fig. 2J), suggesting that TKT may enhance migration and invasion through EMT regulation. Apoptosis analysis by flow cytometry showed that TKT overexpression significantly reduced apoptosis in Huh7 cells, while TKT knockdown increased apoptosis in HCCLM3 cells (Fig. 2H). Under conditions simulating TACE therapy with lobaplatin treatment, cells overexpressing TKT exhibited reduced apoptosis, indicating that TKT may confer survival advantages in the TACE microenvironment by suppressing apoptosis. Finally, cell proliferation under lobaplatin-induced stress was assessed using the CCK-8 assay. Lobaplatin suppressed proliferation in both Huh7 and HCCLM3 cells, but proliferation was considerably higher in the TKT-overexpression group compared to the TKT-knockdown group (Fig. 2I). Notably, TKT overexpression enhances the ability of forming tumoursphere structures in Huh7 cells (Fig. 2K). Collectively, these findings demonstrate that TKT enhances tumorigenic potential in HCC cells by regulating proliferation, migration, invasion, EMT, and apoptosis, thereby promoting cell survival and growth under TACE treatment conditions.

### TKT interacts with RAF1 and activates the ERK pathway

**Fig. 3:**
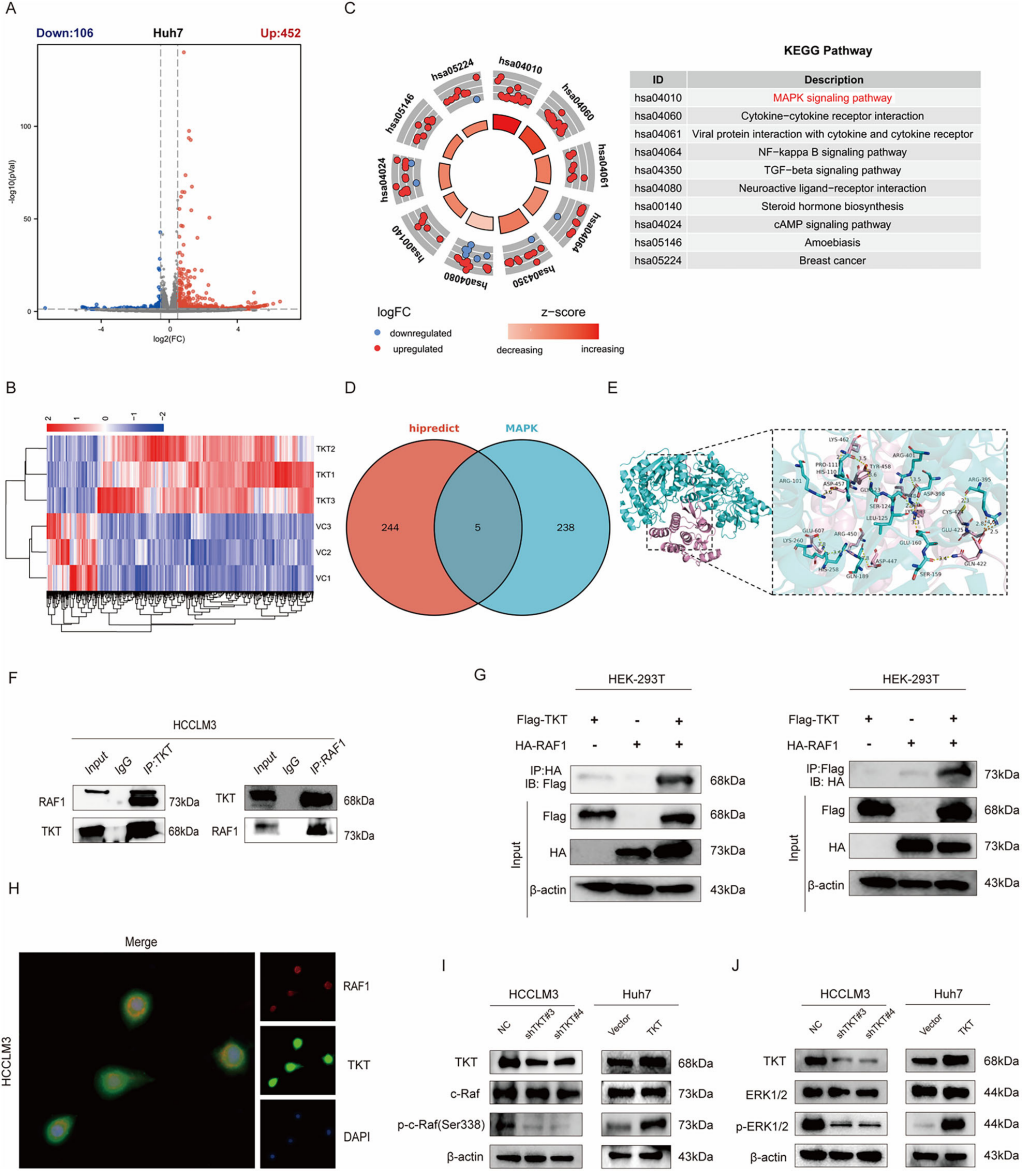
TKT interacts with RAF1 and activates ERK signaling. **A**, **B** Volcano plot and heatmap of DEGs upon TKT overexpression. **C** KEGG analysis highlighting MAPK signaling. **D**, **E** Venn diagram and molecular docking predicting TKT–RAF1 interaction. **F**–**H** Interactions between endogenous and exogenous TKT and RAF1, and immunofluorescence confirming cytoplasmic co-localization. **I**, **J** Western blot showing TKT regulates c-Raf (Ser338) and ERK1/2 phosphorylation.

To interrogate the potential molecular mechanism of TKT in HCC, RNA-seq analysis was performed on Huh7 cells overexpressing TKT in comparison with control cells. In aggregate, 558 differentially expressed genes (DEGs) were identified—452 upregulated and 106 downregulated (Fig. 3A, B). KEGG enrichment analysis indicated that the MAPK signaling pathway was the most significantly altered in response to TKT overexpression (Fig. 3C). GO enrichment analysis showed that TKT overexpression preferentially upregulated genes governing signal transduction, cell motility, and extracellular matrix organization (Supplementary Fig. S2A). These patterns suggest that TKT enhances intercellular communication and migration, thereby increasing cellular activity and responsiveness to external stimuli and potentially driving cellular remodeling and tumor invasion. Using the Hipredict online database to predict TKT-interacting proteins within the MAPK pathway, five candidate genes were identified, with RAF1 being the most prominent (Fig. 3D, Supplementary Fig. S2B). GSEA enrichment indicates upregulation of pathways related to keratinization, cytoskeletal remodeling, and H4 histone acetyltransferase activity, whereas sphingolipid metabolism is downregulated. These alterations are consistent with hallmarks of hepatocellular carcinoma progression, including heightened proliferation and migration together with epigenetic activation (Supplementary Fig. S2C). Molecular docking simulations based on the protein structures of TKT and RAF1 further indicated a strong potential for their interaction (Fig. 3E). Co-immunoprecipitation (CoIP) experiments confirmed endogenous interaction between TKT and RAF1 in HCCLM3 cells (Fig. 3F). To further validate the TKT–RAF1 interaction, we performed reciprocal exogenous Co-IP in HEK-293T cells co-expressing TKT-Flag and RAF1-HA. Anti-Flag immunoprecipitation followed by HA immunoblotting detected RAF1-HA in the TKT-Flag precipitate, and reciprocal anti-HA immunoprecipitation followed by Flag immunoblotting detected TKT-Flag in the RAF1-HA precipitate (Fig. 3G). Immunofluorescence assays demonstrated co-localization of TKT and RAF1, predominantly within the cytoplasm of HCCLM3 cells (Fig. 3H). Functionally, inhibition of TKT in HCCLM3 cells reduced p-c-Raf (Ser338), whereas overexpression of TKT in Huh7 cells increased p-c-Raf (Ser338) levels. Total c-Raf expression remained unchanged (Fig. 3I). Similarly, TKT inhibition decreased downstream p-ERK1/2 levels, while TKT overexpression led to their upregulation (Fig. 3J). These findings collectively indicate that TKT directly binds to RAF1 and facilitates activation of the MAPK/RAF1/ERK signaling cascade.

### TKT enhances c-Myc protein stability via ERK pathway activation

**Fig. 4:**
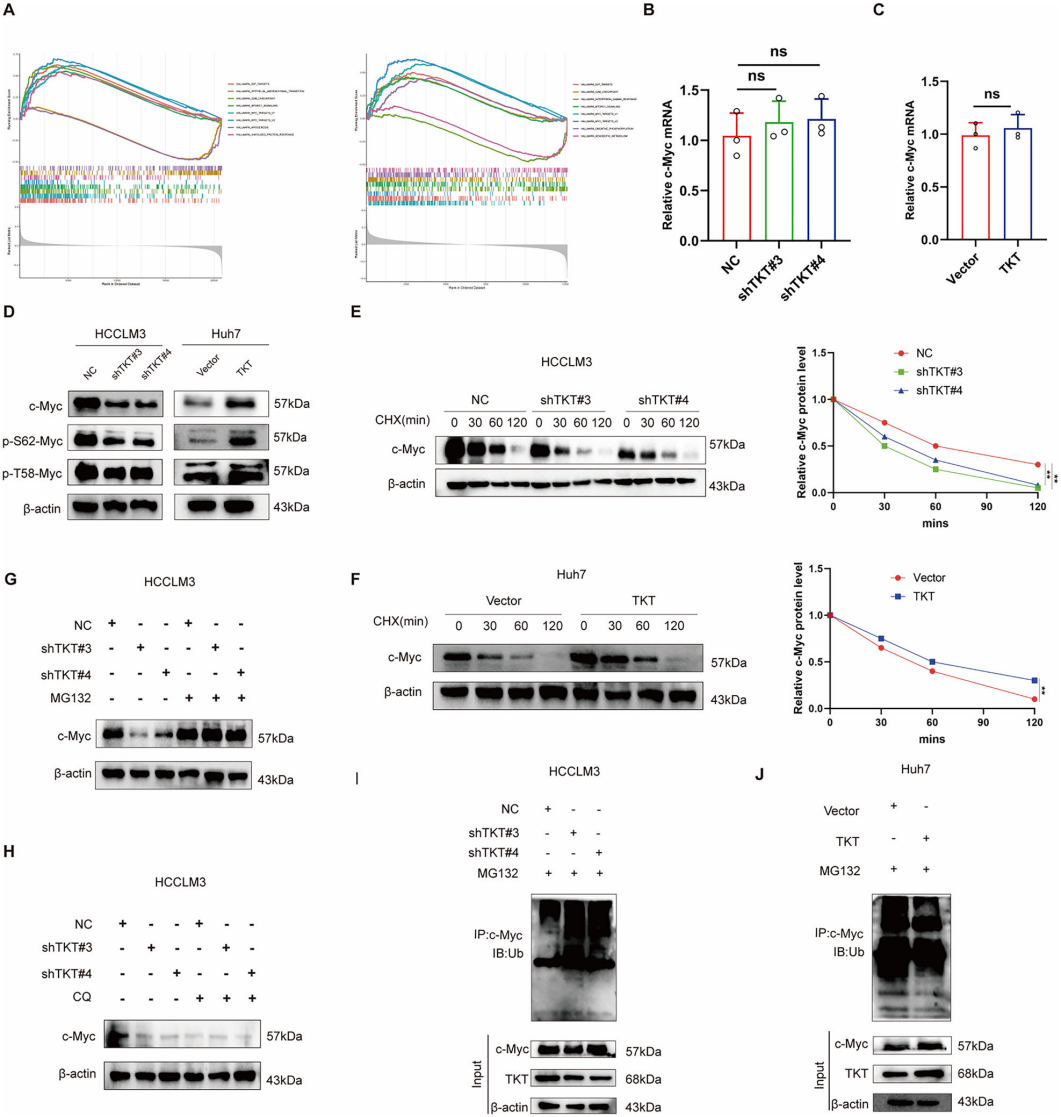
TKT stabilizes c-Myc by activating ERK and suppressing ubiquitin-mediated degradation. **A** GSEA showing enrichment of Myc targets in TKT-high HCC. **B**, **C** qRT-PCR showing TKT does not alter c-Myc mRNA. **D** Western blot showing TKT regulates c-Myc and p-S62-c-Myc protein. **E**, **F** CHX chase assays showing accelerated c-Myc degradation upon TKT knockdown and delayed degradation upon overexpression. **G**, **H** MG132, but not chloroquine, rescues c-Myc in TKT-deficient cells. **I**, **J** Ubiquitination assays showing TKT knockdown enhances, while overexpression reduces, c-Myc ubiquitination.

The ERK pathway regulates c-Myc stability through direct phosphorylation at serine 62 (S62), a mechanism that has been well established. Gene set enrichment analysis (GSEA) of the GSE104580 and GSE14520 datasets revealed that Myc target genes were significantly enriched in the TKT high-expression HCC group (Fig. 4A). Based on this observation, we hypothesized that TKT enhances ERK-mediated stabilization of c-Myc through interaction with RAF1. Experimental results demonstrated that TKT does not regulate c-Myc at the mRNA transcriptional level, as confirmed by qRT-PCR (Fig. 4B, C). In TKT-knockdown HCCLM3 cells, both total c-Myc and p-c-Myc(S62) levels were significantly reduced in parallel with decreased p-ERK1/2. Conversely, in Huh7 cells overexpressing TKT, c-Myc and p-c-Myc(S62) levels were markedly elevated (Fig. 4D). Cycloheximide (CHX) chase assays revealed that TKT knockdown significantly accelerated c-Myc degradation compared with control cells (Fig. 4E). Pretreatment with the proteasome inhibitor MG132 effectively rescued c-Myc degradation caused by TKT deficiency, whereas the lysosomal inhibitor CQ had no effect (Fig. 4G, H). Conversely, TKT overexpression significantly delayed c-Myc degradation in Huh7 cells (Fig. 4F). Ubiquitination analysis further confirmed that TKT knockdown enhanced c-Myc ubiquitination and degradation, while TKT overexpression suppressed these processes (Fig. 4I, J). Collectively, these results support the conclusion that TKT stabilizes c-Myc protein by interacting with RAF1 and activating the ERK pathway, thereby inhibiting ubiquitin-mediated degradation and increasing c-Myc protein levels.

### c-Myc promotes TKT-mediated progression of HCC

**Fig. 5:**
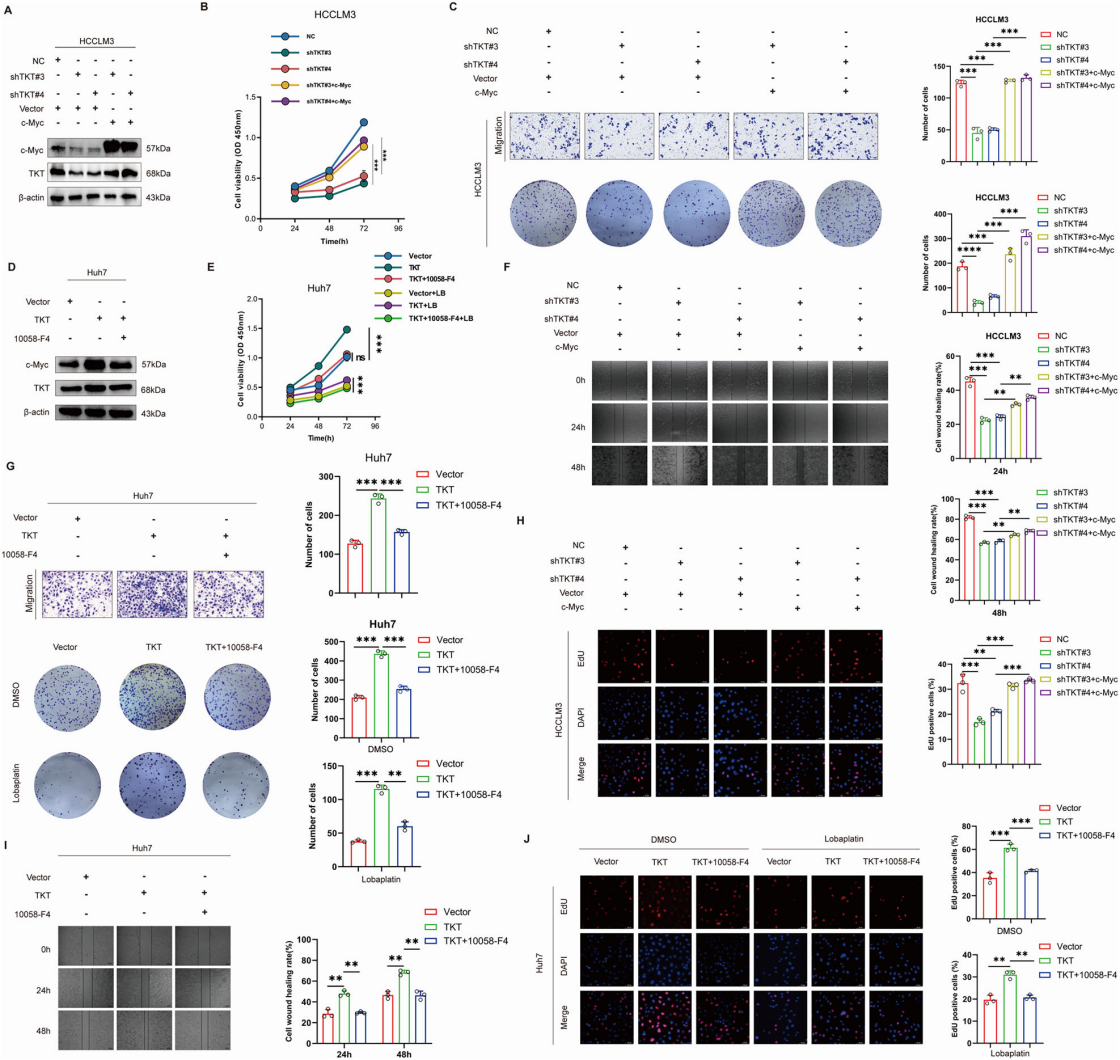
c-Myc mediates TKT-driven HCC progression. **A**–**C** Rescue of proliferation, invasion, and colony formation in TKT-knockdown HCCLM3 cells by c-Myc overexpression. **D**, **E** Western blot and CCK-8 assays showing 10058-F4 suppresses c-Myc and abolishes TKT-driven proliferation in Huh7 cells. **F**–**H** Wound healing, Transwell, and colony assays showing c-Myc rescues or 10058-F4 suppresses TKT-driven migration and invasion. **I**, **J** EdU assays showing c-Myc overexpression restores proliferation suppressed by TKT knockdown, while 10058-F4 blocks TKT-induced proliferation.

To evaluate whether c-Myc is implicated in the progression of HCC mediated by TKT, c-Myc was overexpressed in TKT-silenced HCCLM3 cells, which restored TKT expression (Fig. 5A). Restoration of c-Myc expression reversed the inhibitory effects of TKT knockdown on HCCLM3 cell proliferation and migration (Fig. 5B, C, F, H). Conversely, in TKT-overexpressing Huh7 cells, treatment with the c-Myc inhibitor 10058-F4 effectively abolished the pro-proliferative and pro-migratory effects of TKT overexpression (Fig. 5D, E, G, I, J). Taken together, these results suggest that c-Myc is essential for driving the progression of TKT-induced HCC.

### c-Myc promotes transcription of TKT in HCC

**Fig. 6:**
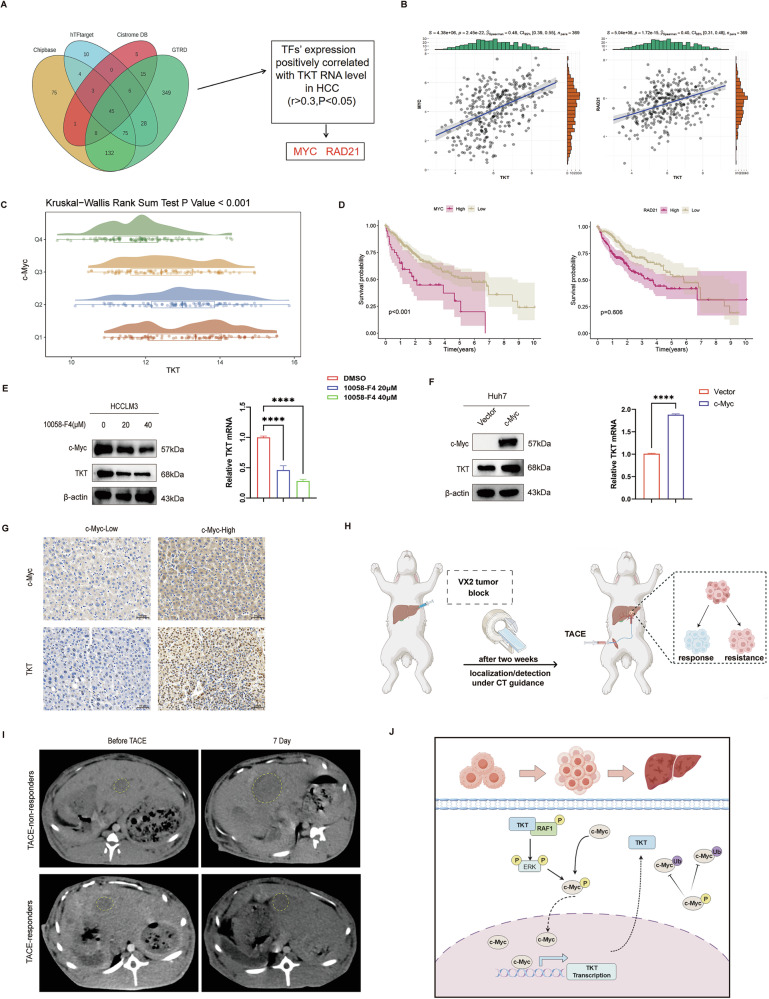
c-Myc transcriptionally regulates TKT and correlates with TACE resistance in vivo. **A**, **B** Bioinformatic prediction and correlation analysis identifying c-Myc as a TKT regulator. **C**, **D** Kruskal–Wallis and Kaplan–Meier analyses showing higher TKT expression and poorer survival with high c-Myc. **E**, **F** Western blot and qRT-PCR showing 10058-F4 reduces, while c-Myc overexpression increases, TKT levels. **G** IHC showing higher TKT expression in c-Myc–high tumors. **H** VX2 rabbit TACE model. **I** Tumor CT images before and after TACE. **J** The mechanism chart.

To gain a deeper understanding of the regulatory role of TKT in HCC development, bioinformatics analyses across four public databases identified 45 candidate transcription factors, including c-Myc and RAD21 (Fig. 6A). mRNA correlation analysis using the TCGA-LIHC dataset revealed that only c-Myc and RAD21 showed positive correlations with TKT expression (*r* > 0.3, *P* < 0.05, Fig. 6B). The survival analysis revealed a significant correlation between high c-Myc expression and shorter overall survival (OS), whereas RAD21 showed no statistical significance (Fig. 6C). Moreover, when patients were stratified into quartiles (Q1–Q4) based on MYC expression, TKT expression levels increased progressively with higher MYC expression (Fig. 6D), suggesting that MYC may act as a transcription factor positively regulating TKT expression. Western blotting further confirmed that c-Myc inhibition markedly reduced TKT expression at both protein and mRNA levels, whereas c-Myc overexpression led to a significant upregulation of TKT (Fig. 6E, F). Immunohistochemistry (IHC) staining of specimens from TACE-resistant patients showed that TKT expression was lower in c-Myc–low cases, whereas c-Myc–high cases exhibited higher TKT expression (Fig. 6G). As illustrated in Fig. 6H, a VX2 rabbit orthotopic liver cancer TACE model was established. Serial CT scans collected from tumor sites at 7 days revealed slower tumor progression in the TACE-responders group, whereas tumors in the TACE-non-responders group progressed rapidly (Fig. 6I). Subsequent IHC results showed higher expression of TKT and c-Myc, as well as increased proliferation and reduced apoptosis in the TACE non-responders group, consistent with our previous findings (Supplementary Fig. S2D).

## Discussion

Our goal in this study was to explore how TKT contributes to HCC progression and its impact on resistance to TACE therapy, while delineating the underlying molecular mechanisms. Our principal findings establish that TKT is overexpressed in TACE-resistant HCC, where it forms a positive feedback loop with c-Myc via RAF1-mediated ERK activation, thereby promoting tumor proliferation, migration, EMT, and apoptosis resistance, highlighting a novel metabolic-oncogenic circuit driving therapeutic refractoriness.

The increased expression of TKT in TACE-resistant HCC patients, revealed by weighted gene co-expression network analysis and machine learning algorithms, highlights its importance as a central gene in hypoxia-driven metabolic reprogramming. Under TACE-induced ischemic stress, tumor cells exploit the PPP to sustain nucleotide biosynthesis and redox balance, with TKT acting as a rate-limiting enzyme in the non-oxidative branch. Our in vitro functional assays demonstrate that TKT overexpression enhances proliferative capacity, as evidenced by increased EdU incorporation and colony formation, while knockdown impairs these processes. This aligns with the hypothesis that TKT confers adaptive survival advantages by rerouting glucose metabolism away from oxidative phosphorylation toward anabolic pathways, mitigating oxidative stress and supporting rapid cell division in nutrient-deprived microenvironments.

Further mechanistic insights reveal that TKT interacts directly with RAF1, leading to phosphorylation at Ser338 and subsequent activation of the ERK pathway. RNA-seq and KEGG enrichment analyses confirmed MAPK signaling as the predominant pathway altered by TKT modulation, with co-immunoprecipitation and immunofluorescence validating the cytoplasmic co-localization of TKT and RAF1. This interaction likely stabilizes RAF1 conformation, amplifying downstream ERK phosphorylation and integrating metabolic cues with mitogenic signaling. Consequently, activated ERK phosphorylates c-Myc at Ser62, enhancing its protein stability and inhibiting ubiquitin-mediated degradation, as corroborated by cycloheximide chase and ubiquitination assays. The resultant c-Myc accumulation drives oncogenic transcription, perpetuating tumor aggressiveness in the context of TACE resistance.

TKT catalyzes key reactions in the non-oxidative branch of the PPP. By directing carbon flux through this branch, TKT enables tumor cells to meet diverse metabolic demands under varying conditions [17]. TKT expression is also closely linked to chemoresistance [18]. Thus, TKT is a critical contributor to tumorigenesis and progression. Our findings are consistent with prior reports linking TKT overexpression to poor HCC prognosis and tumor promotion [12]. For instance, TKT nuclear localization enhances HCC proliferation via metabolic and non-metabolic functions, mirroring our observations of TKT’s role in cell cycle progression and viability [13]. Across multiple cancers, including HCC, TKT upregulation correlates with increased PPP flux and stress-adaptive survival [19], aligning with our identification of TKT as a hypoxia-related gene associated with TACE resistance. Important differences also emerge. Previous work showed that TKT-mediated inhibition of the farnesoid receptor facilitates HCC initiation [20], whereas the present data delineate a RAF1–ERK–c-Myc signaling axis, addressing a gap in understanding non-metabolic signaling by TKT.

Aberrant glycolysis and lipid metabolism are increasingly recognized as key drivers of treatment refractoriness in HCC and plausibly contribute to TACE failure [21]. AKR1B1 inhibition reverses systemic therapy resistance by targeting metabolic plasticity in HCC, supporting the hypothesis that perturbing PPP enzymes can sensitize tumors to locoregional therapies [22]. We extend this framework by integrating oncogenic signaling rather than attributing resistance solely to mitochondrial adaptations [23, 24]. We identify a feedback loop that amplifies metabolic and transcriptional programs, providing a mechanistic basis for the heterogeneous outcomes observed across clinical TACE cohorts [25].

c-Myc is a key regulatory factor involved in various biological processes, primarily through the direct or indirect modulation of transcription factors that control the expression of multiple genes [26]. The relationship between TKT and c-Myc has been observed in other cancer types as well. For example, in MYC-driven medulloblastomas, MYC upregulates TKT transcriptionally, enhancing the PPP to support cell proliferation and mitigate oxidative stress [27]. Additionally, in head and neck squamous cell carcinoma, the c-MYC-NRF2 axis directly activates TKT, promoting malignant progression [28]. In the present study, through bioinformatics analysis and experimental validation, we identified that c-Myc binds to the TKT promoter region, enhancing its transcriptional activity. These findings suggest that TKT promotes c-Myc expression via the MAPK/ERK pathway. In turn, the accumulation of c-Myc feeds back to further enhance TKT expression, forming a positive feedback loop that drives HCC tumorigenesis.

Despite these contributions, limitations warrant consideration. The reliance on cell lines and retrospective clinical samples may limit generalizability to heterogeneous HCC populations, potentially overlooking inter-patient variability in etiological factors like viral hepatitis. Sample size in the TACE cohort was modest, and while machine learning mitigated bias, prospective validation in larger multicenter trials is needed. Methodological constraints, such as simulating TACE via lobaplatin without full emulation of embolization-induced hypoxia, could underestimate in vivo complexities; incorporating organoid models or advanced hypoxia chambers in future studies would address this.

## Conclusion

In summary, this research reveals a novel mechanism whereby the TKT-c-Myc feedback loop accelerates HCC progression (Fig. 6J). Targeting both TKT and c-Myc may offer a promising therapeutic strategy for patients with TACE resistance.

## Methods

### Collection of clinical specimens

Liver cancer samples of tissue were obtained from individuals undergoing TACE treatment at the Affiliated Hospital of Nantong University (Nantong, Jiangsu, China) during January 2018 to December 2023. Everyone involved was granted informed consent before their involvement in the study, which was approved by the Ethics Committee of the Affiliated Hospital of Nantong University (approval number: 2023-L038). The research project rigorously complied with the guidelines established in the Declaration of Helsinki.

### Cell culture and transfection

Human hepatocellular carcinoma cell lines (Hep1, HepG2, HCCLM3, Huh7, and SNU449) and the HEK293T cell line were obtained from Zhongqiao Xinzhou Biotechnology (Shanghai, China). Cells were maintained in DMEM High Glucose (Biochannel, China) supplemented with fetal bovine serum (FBS; Vazyme Biotechnology, China) and penicillin/streptomycin (Vazyme Biotechnology, China) in a humidified incubator at 37 °C with 5% CO₂. The TKT expression plasmid (pTSB02-GFP-PURO) and TKT-targeting shRNAs were developed and synthesized by Quanyang Biotechnology (Shanghai, China); The shRNA sequences can be found in Supplementary Table S1. For transfection, cells were seeded in six-well plates (5 ×10⁵ per well) and transfected with the specified plasmids using Neofect Transfection Reagent (Biowell, China), following the manufacturer’s guidelines, and then subjected to selection as outlined.

### Cell viability assay

The CCK-8 Cell Proliferation and Cytotoxicity Assay Kit (A311-01, Vazyme) was used to evaluate cell viability. The HCC cells were seeded in 96-well plates at a density of 1.5× 10³ cells/well and maintained for 24, 48, or 72 h. Following incubation with CCK-8 reagent at 37 °C for 2 h, the optical density (OD) was measured at 450 nm.

### Colony formation assay

A total of 500 cells were seeded into individual wells of 6-well plates and cultured for 2 weeks. Cells were fixed with 4% paraformaldehyde, stained with crystal violet (C0121, Beyotime).

### Transwell and wound-healing assays

Transwell migration was evaluated using chambers equipped with 8-μm pore inserts (Corning). The lower chamber was filled with medium containing 10% FBS, and 3 ×10⁴ cells in 200 μL suspension were placed in the upper insert. After 48 h, migrated cells were fixed with 4% PFA for 30 min, stained with crystal violet, and subsequently imaged and quantified. For wound-healing assays, confluent monolayers were established in 6-well plates, and linear scratches were generated using a 10 μL pipette tip. Wound closure was recorded at predefined intervals to assess migratory capacity.

### Apoptosis analysis

Apoptosis was assessed using the Annexin V–FITC Apoptosis Detection Kit (Beyotime) in accordance with the manufacturer’s instructions. Samples were analyzed by flow cytometry.

### Western blotting

Following SDS-PAGE separation, samples of proteins were put onto PVDF membranes (Millipore, USA). Primary antibodies were used to incubate the membranes for an entire night at 4 °C after they had been blocked for two hours with 5% non-fat milk. Following a wash, the membranes were incubated for two hours at room temperature with HRP-conjugated secondary antibodies (Jackson ImmunoResearch, Cat#111-035-003 and Cat#115-035-003; 1:2000). Applying an enhanced chemiluminescence (ECL) detection reagent, protein bands were evident. The antibodies were listed in Supplementary Table 2.

### Immunohistochemistry

After being dewaxed in xylene, specimens of tissue were rehydrated using a graded series of ethanol washes with progressively lower concentrations. The slides were then blocked with 5% BSA after being sterilized with a 0.3% hydrogen peroxide solution to suppress endogenous peroxidase activity. Slides were kept at 4 °C for the entire night in primary antibodies, further coated with a streptavidin-biotin complex following TBST washing, and 3,3′-diaminobenzidine (DAB) was used for imaging.

### Immunofluorescence assay

The hepatocellular carcinoma cells were fixed in 4% paraformaldehyde for 30 min, treated with 0.1% Triton X-100 for 10 min to permeabilize, and blocked with 5% BSA for 1 h. The primary antibodies against TKT (Proteintech, Cat#11039-1-AP) and RAF1 (Santa Cruz Biotechnology, Cat#sc-7267) were incubated overnight at 4 °C. Following washes with TBST, the cells were treated with the corresponding fluorescent secondary antibodies, and fluorescence images were captured using a fluorescence microscope.

### Cycloheximide chase assay

Cells were exposed to 50 µg/mL cycloheximide (CHX) for the indicated times to evaluate protein degradation. Proteins were extracted with RIPA lysis buffer and analyzed by Western blotting; densitometric quantification was performed using ImageJ.

### Ubiquitination assay

Log-phase cells were seeded in 10-cm dishes and cultured for 24 h, then treated with 10 µM MG-132 for 8 h. Cell lysates were incubated overnight at 4 °C with primary antibodies, followed by a 3-h incubation with Protein A/G agarose beads to immunoprecipitate antibody–protein complexes. Ubiquitination of c-Myc was assessed by Western blotting using an anti-Ub antibody.

### RT-qPCR and RNA sequencing

The primer sequences used for amplification are provided in Supplementary Table S3.

For RNA sequencing, total RNA was extracted from TKT-overexpressing HCCLM3 cells and their control counterparts using TRIzol reagent. Transcriptome sequencing and analysis were performed by OBiO Technology Corp. Ltd. (Shanghai, China). Library quality control was verified by quantifying DNA concentration on a Qubit fluorometer and evaluating fragment-size distributions using an Agilent Bioanalyzer. Libraries that met quality criteria were sequenced as 150-bp paired-end reads (PE150) on the Illumina NovaSeq 6000 platform. Raw reads were processed with fastp (v0.22.0) to remove low-quality sequences, yielding clean reads, which were subsequently aligned to the reference genome with HISAT2 (v2.1.0). Gene expression was summarized as FPKM values, and gene-level read counts were obtained via featureCounts.

### Animal study

Six to eight-month-old New Zealand white rabbits, sourced from the Animal Center of Nantong University, were randomly assigned to experimental groups. To establish the liver cancer TACE model, VX2 tumor fragments were injected into the left hepatic lobe under CT guidance. All animal experimental procedures were reviewed and approved by the Animal Experiment Ethics Committee of Nantong University (Approval No. P20230221-017). Investigators were not blinded to group allocation during treatment administration; however, outcome assessment was performed using standardized objective criteria.

### Sample size determination

The animal sample size (*n* = 3 per group) was determined based on previous literature and pilot experiments evaluating tumor response in the VX2 rabbit model. No formal power calculation was performed.

### Statistical analysis

All experiments were independently repeated at least three times unless otherwise stated. Data are presented as mean ± standard deviation (SD). Statistical analyses were performed using GraphPad Prism 9.0 (GraphPad Software, USA). Comparisons between two groups were conducted using unpaired two-tailed Student’s *t* test. For multiple group comparisons, one-way ANOVA followed by Tukey’s post hoc test was used. Normality of data distribution was assessed using the Shapiro–Wilk test. Variance similarity between groups was evaluated before applying parametric tests. A two-sided *P* value < 0.05 was considered statistically significant. For RNA sequencing analysis, differential expression was performed using DESeq2. Genes with |log_2_ fold change|> 1 and adjusted *P* value < 0.05 were considered significantly differentially expressed.

## Supplementary information

Supplementary information

Original western blots

Supplementary Table S1

Supplementary Table S2

Supplementary Table S3

## Data Availability

The RNA sequencing data generated in this study have been deposited in the Gene Expression Omnibus (GEO) database under accession number GSE322668.
