# Correction: A positive feedback loop between TKT and c-Myc drives TACE resistance in hepatocellular carcinoma

**DOI:** 10.1038/s41420-026-03216-6

**Published:** 2026-07-15

**Authors:** Yifu Xiao, Mingyu Liu, Ying Zhou, Yunyuan Bao, Guoqing Zhang, Banglong Xu, Wenjie Zheng, Hui Zhao

**Affiliations:** 1 Department of Interventional Radiology, Affiliated Hospital of Nantong University, Medical School of Nantong University, Nantong, China; 2 Research Center of Clinical Medicine, Affiliated Hospital of Nantong University, Medical School of Nantong University, Nantong, China

**Keywords:** Cancer metabolism, Ubiquitylation

Correction to: *Cell Death Discovery* 10.1038/s41420-026-03125-8, published online 21 April 2026

In the accepted manuscript entitled “A positive feedback loop between TKT and c-Myc drives TACE resistance in hepatocellular carcinoma” (Manuscript ID CDDISCOVERY-25-3238R1), we identified three errors in figure presentation. The corrections are described below.

1. Correction to Fig 2D

**Fig 2 original** 
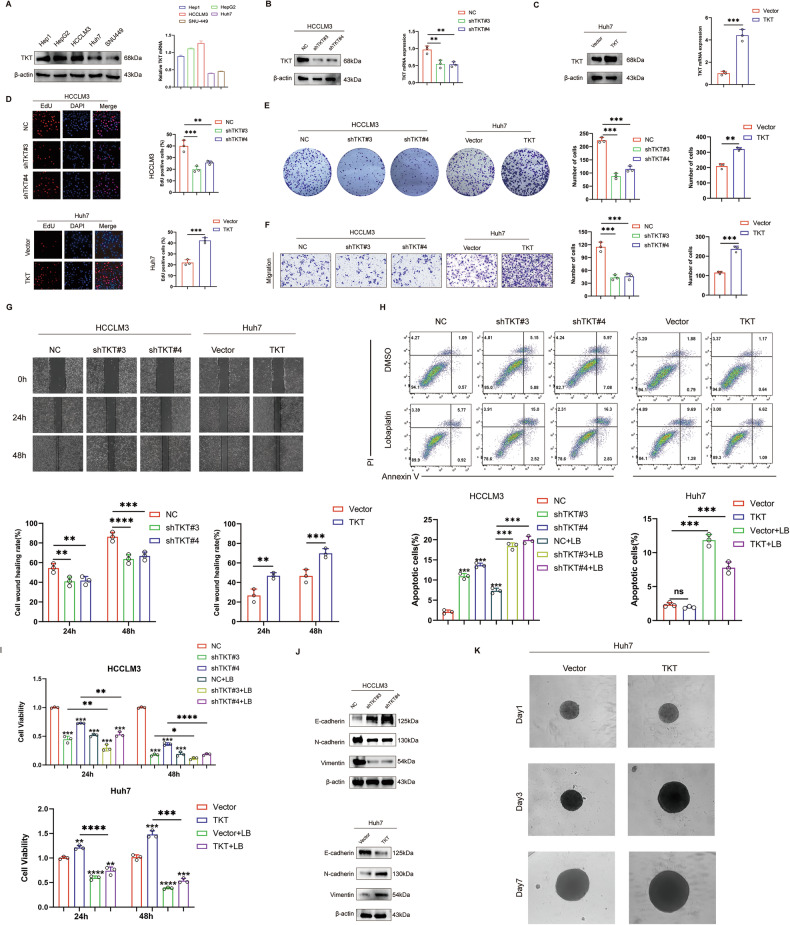


**Fig 2 amended** 
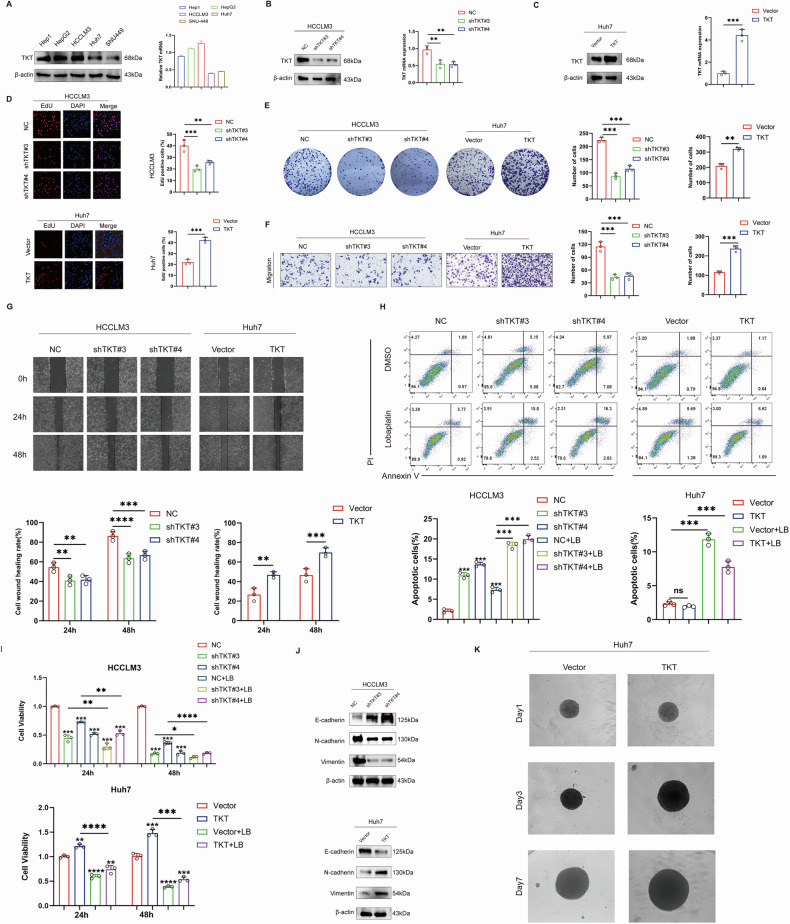


In the Huh7 cell EdU assay shown in Figure 2D, the merged image located in the bottom-right corner, corresponding to the TKT overexpression group, was inadvertently inserted incorrectly. The originally used merged image was not consistent with the corresponding images in the related panels.The correct image has now been provided and should replace the previous one.

2. Correction to Fig 4D

**Fig 4 original** 
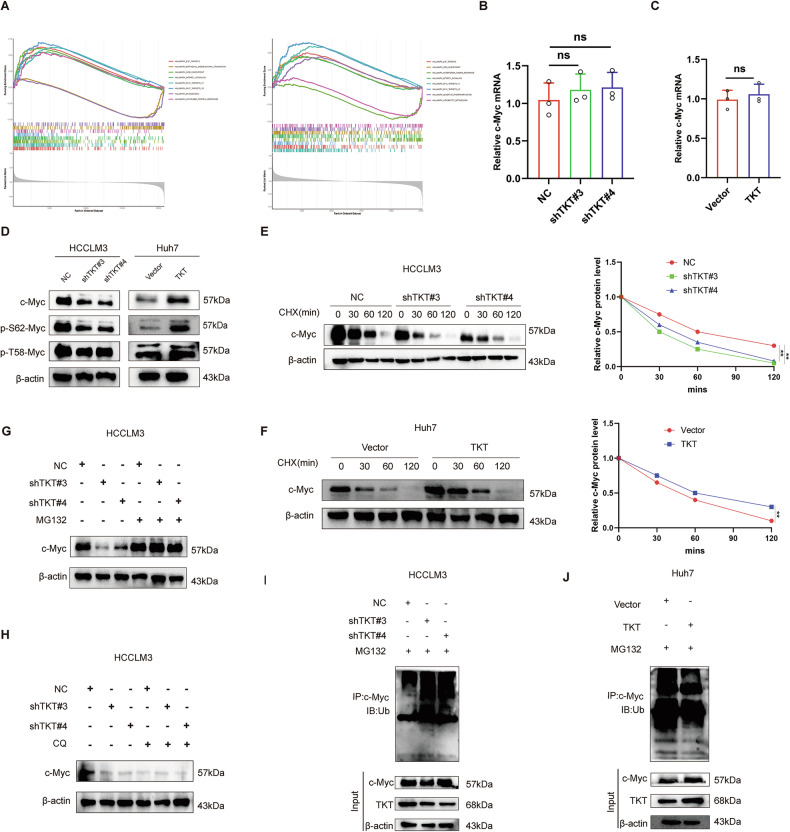


**Fig 4 amended** 
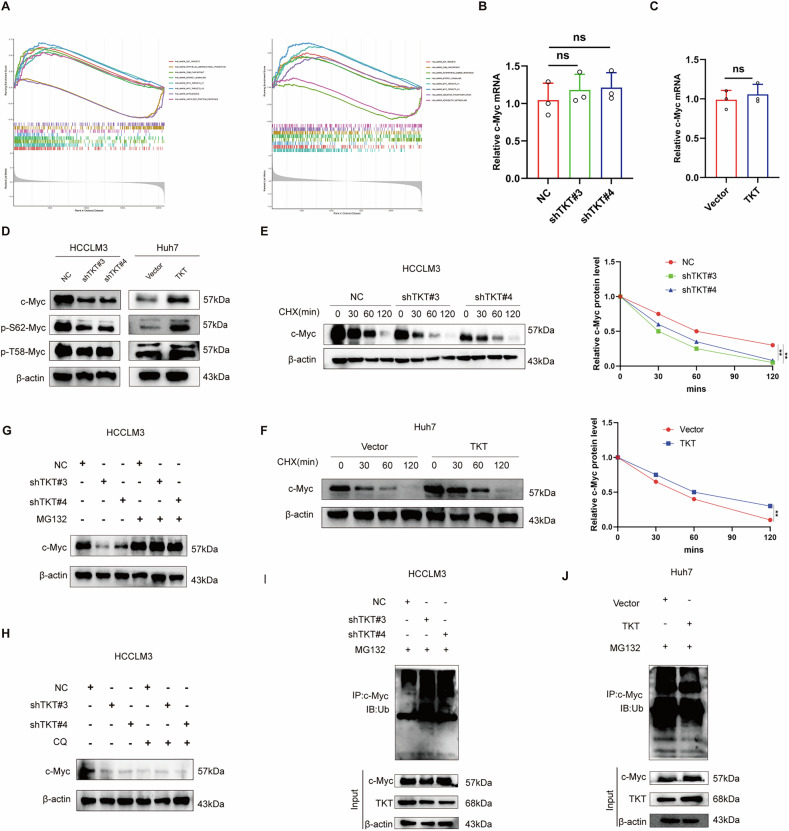


In Fig 4D, the c-Myc band in the first row appeared similar to that in the second row(p-S62-myc). This may have resulted from stripping and re-probing on the same membrane. To ensure scientific rigor, we repeated the experiment and generated a new blot image. The repeated result is consistent with the original findings and does not affect the interpretation of the data or the conclusions of the manuscript.

3. Correction to Fig 5I

**Fig 5 original** 
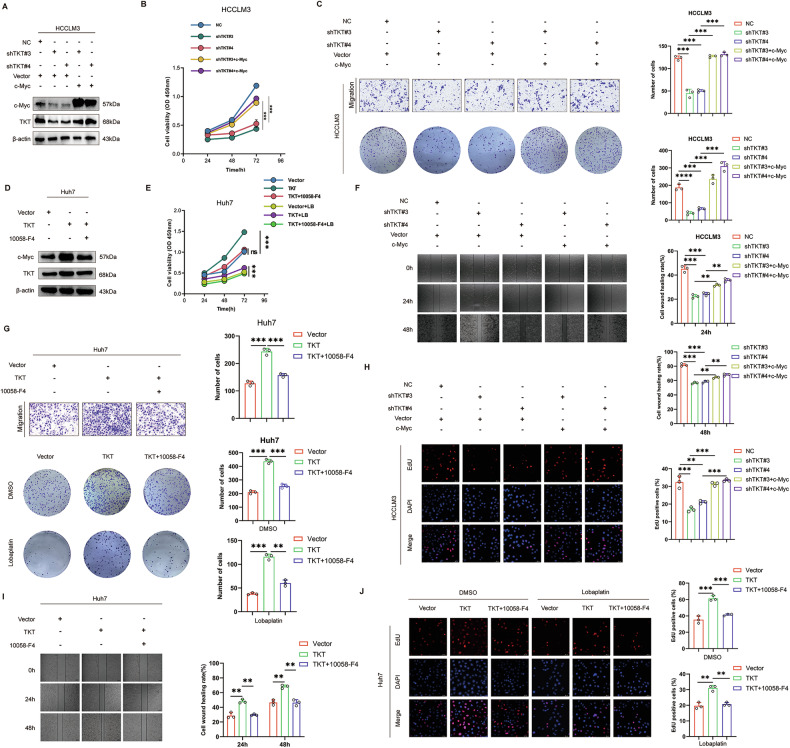


**Fig 5 amended** 
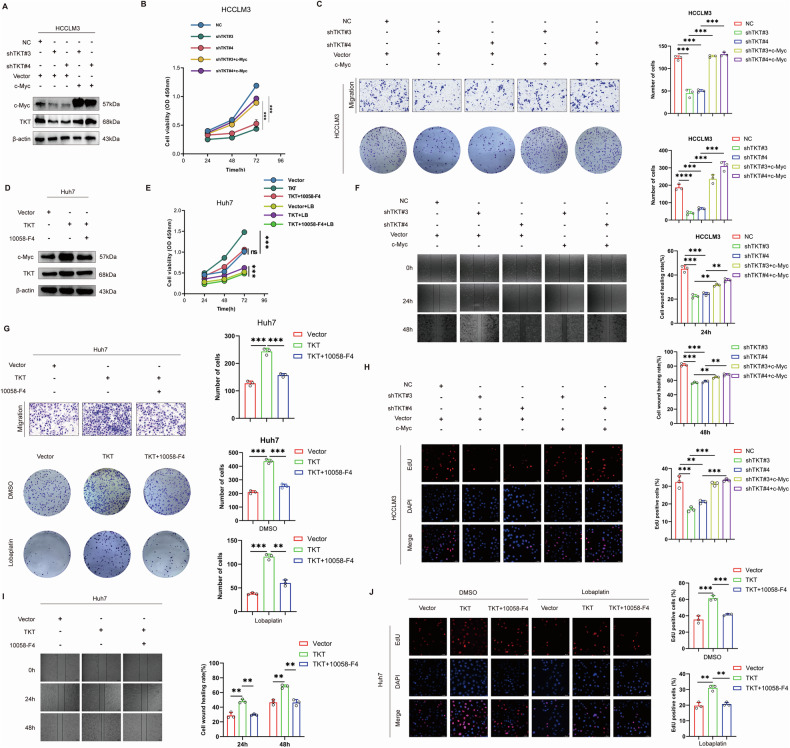


In the Huh7 cell wound-healing assay shown in Figure 5I, the 0 h image located in the upper-right corner, corresponding to the TKT overexpression + 10058-F4 treatment group, was inadvertently used as the image from the preceding group during figure assembly. After reviewing the original files, we confirmed that this was an image-processing error. The image shown below is the correct image.

These corrections do not affect the results, interpretation, or overall conclusions of the study. We sincerely apologize for these errors and for any inconvenience caused.

The original article has been corrected.

## Supplementary information

Original western blots

