# Supplementary material for: A positive feedback loop between TKT and c-Myc drives TACE resistance in hepatocellular carcinoma
Source: Cell Death Discov. 2026 Apr 21;12:267. doi: 10.1038/s41420-026-03125-8 (PMC13233983; doi:10.1038/s41420-026-03125-8)
Supplement: Supplementary file 1 — Supplementary information [file 41420_2026_3125_MOESM1_ESM.docx]

**A positive feedback loop between TKT and c-Myc drives TACE resistance in hepatocellular carcinoma.**

**Supplemental Figures and Figure legends**


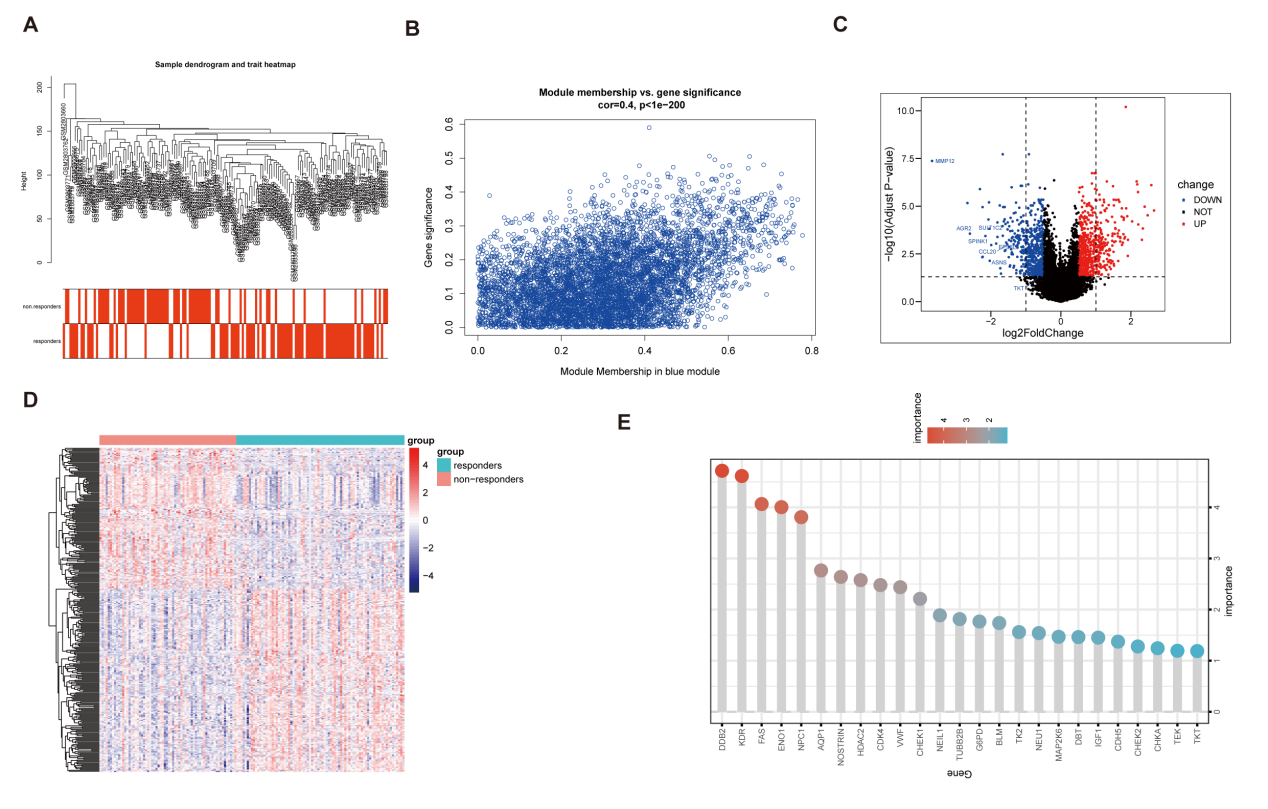


**Figure S1. Supplementary analyses for hub-gene discovery associated with TACE response in HCC.** (A) Sample clustering dendrogram with trait heatmap indicating clinical groups (responders vs. non-responders). (B) Correlation between module membership (MM) and gene significance (GS) within the blue WGCNA module. (C) Volcano plot of differentially expressed genes between responders and non-responders. (D) Heatmap of DEGs. (E) Random-forest ranking of feature importance for candidate hub genes.

**
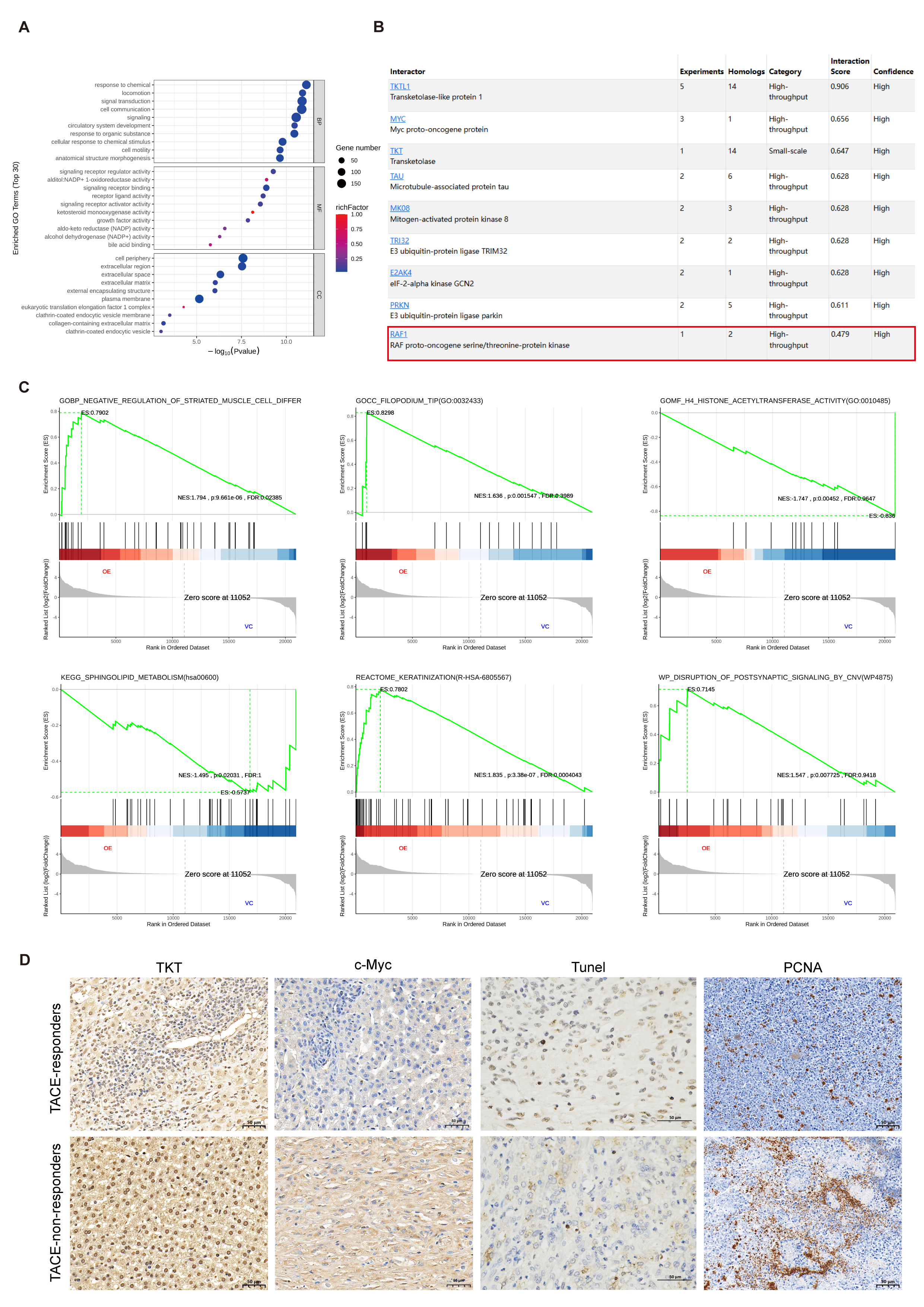
**

**Figure S2. TKT-related functional enrichment, interaction prediction, pathway analysis, and histologic validation.** (A) Gene Ontology enrichment of differentially expressed genes following TKT overexpression. (B) HiPredict-based table of predicted protein–protein interactions involving TKT within the MAPK signaling pathway. (C) Representative GSEA results. (D) IHC shows increased TKT,c-Myc and PCNA staining and reduced TUNEL staining in TACE-non-responders compared with TACE-responders (scale bar, 50 μm).
