# Supplementary material for: Correction: A positive feedback loop between TKT and c-Myc drives TACE resistance in hepatocellular carcinoma
Source: Cell Death Discov. 2026 Jul 15;12:308. doi: 10.1038/s41420-026-03216-6 (PMC13373172; doi:10.1038/s41420-026-03216-6)
Supplement: Supplementary file 1 — Original western blots [file 41420_2026_3216_MOESM1_ESM.pptx]

## Slide 1
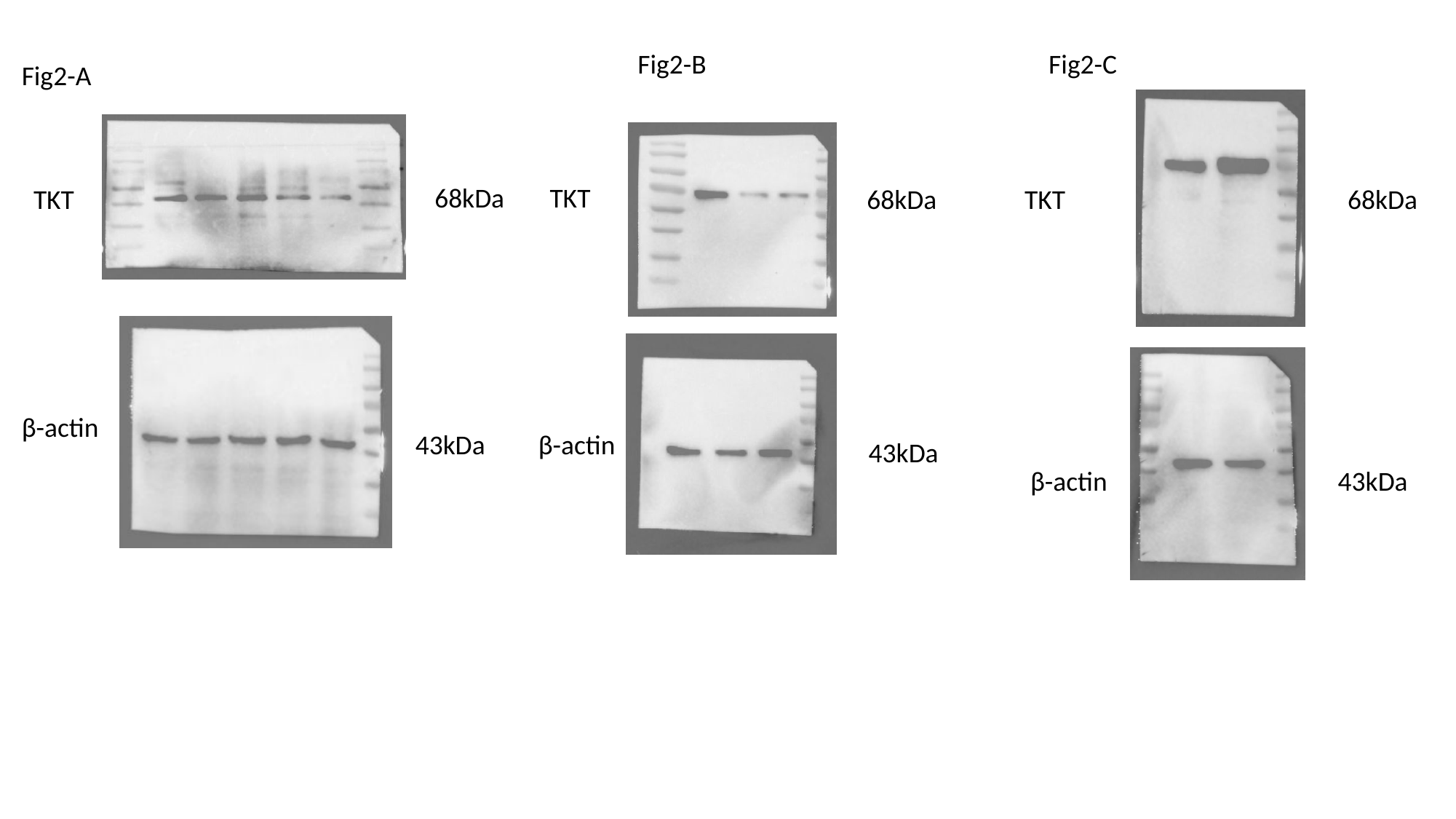

Fig2-B
Fig2-C
Fig2-A
68kDa
TKT
TKT
68kDa
TKT
68kDa
β-actin
43kDa
β-actin
43kDa
β-actin
43kDa

## Slide 2
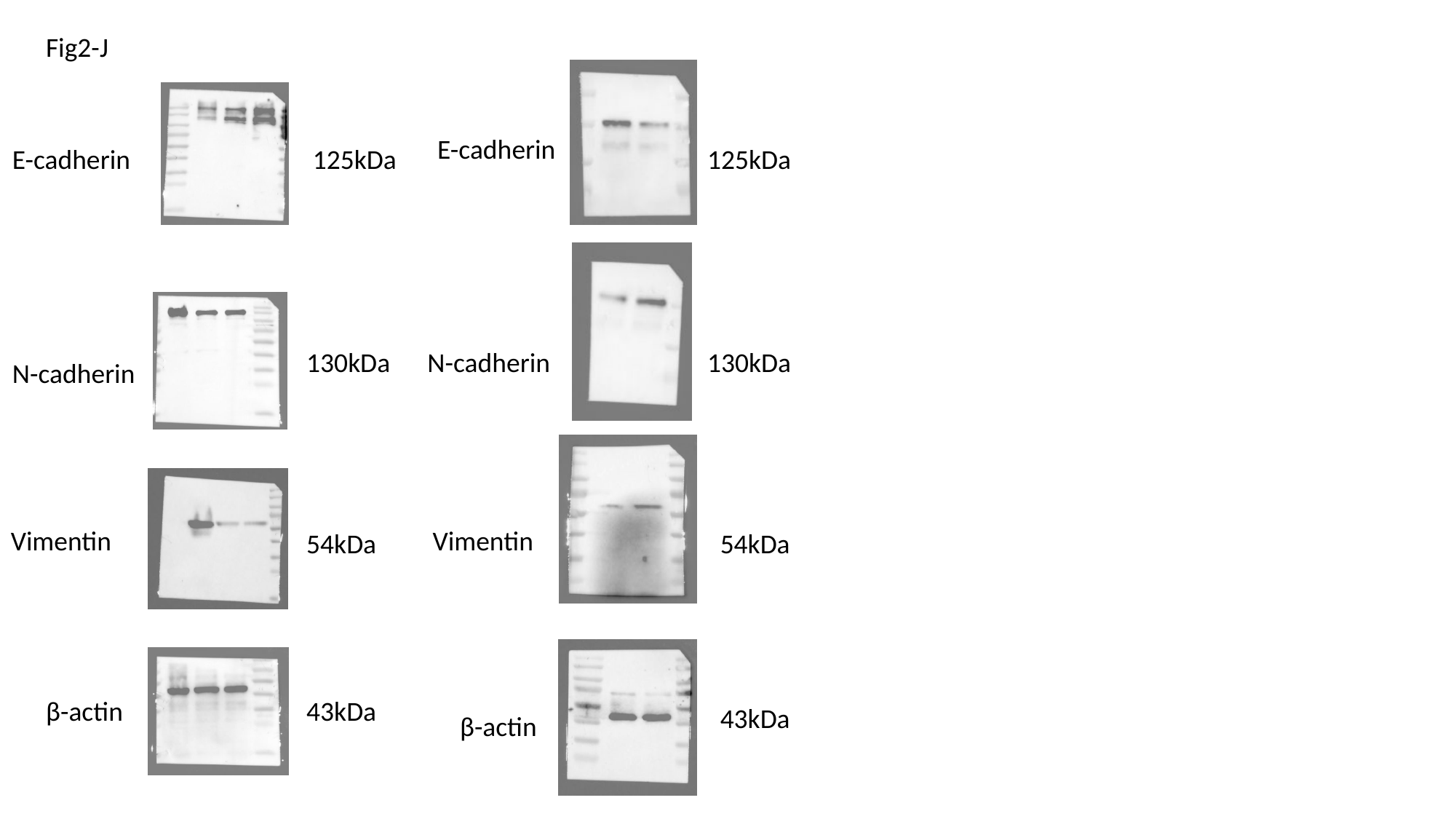

Fig2-J
E-cadherin
125kDa
E-cadherin
125kDa
130kDa
N-cadherin
130kDa
N-cadherin
Vimentin
Vimentin
54kDa
54kDa
β-actin
43kDa
43kDa
β-actin

## Slide 3
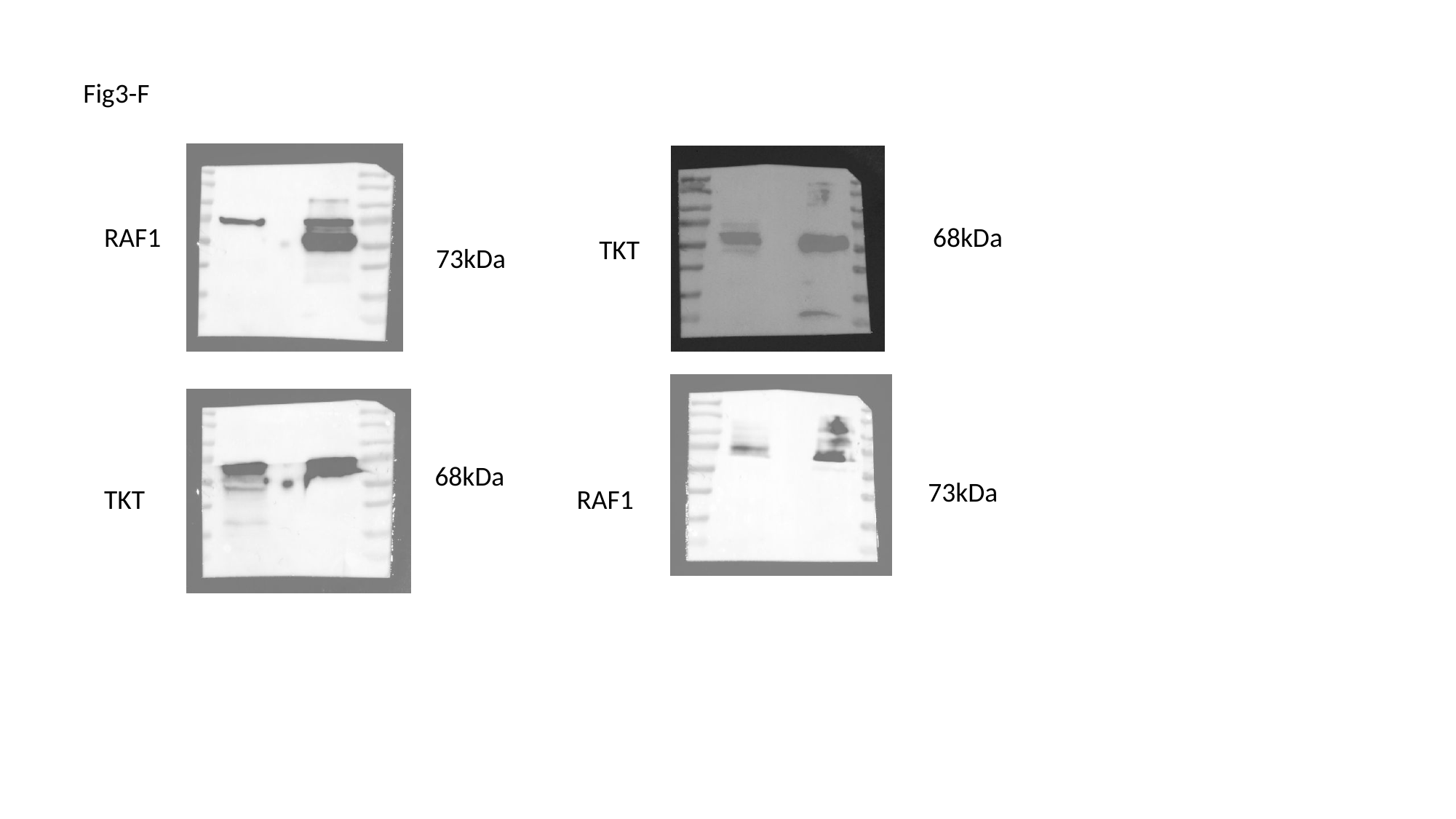

Fig3-F
	73kDa
RAF1
	68kDa
TKT
	68kDa
	73kDa
TKT
RAF1

## Slide 4
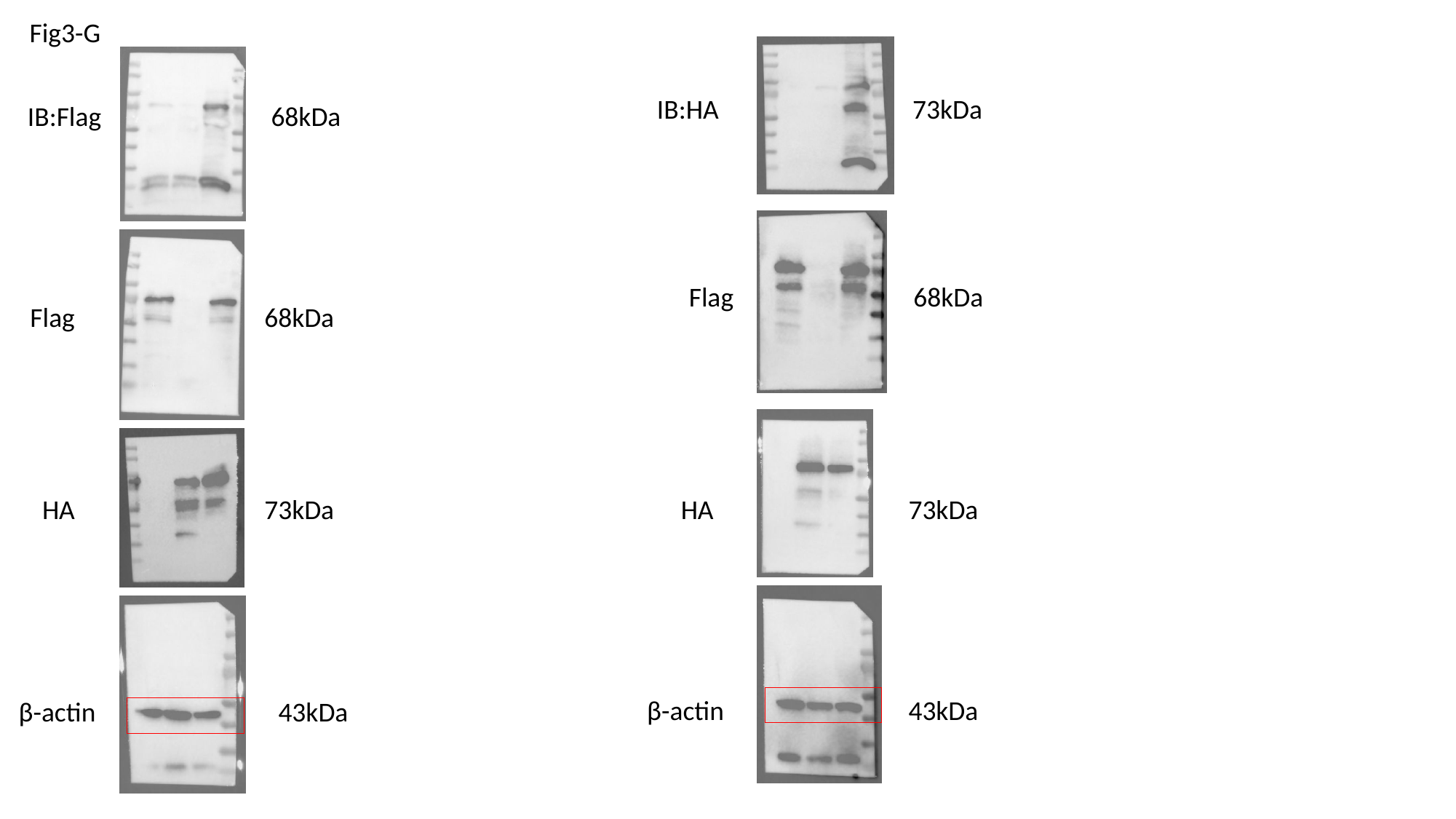

Fig3-G
IB:HA
73kDa
IB:Flag
68kDa
Flag
68kDa
Flag
68kDa
HA
73kDa
HA
73kDa
β-actin
43kDa
β-actin
43kDa

## Slide 5
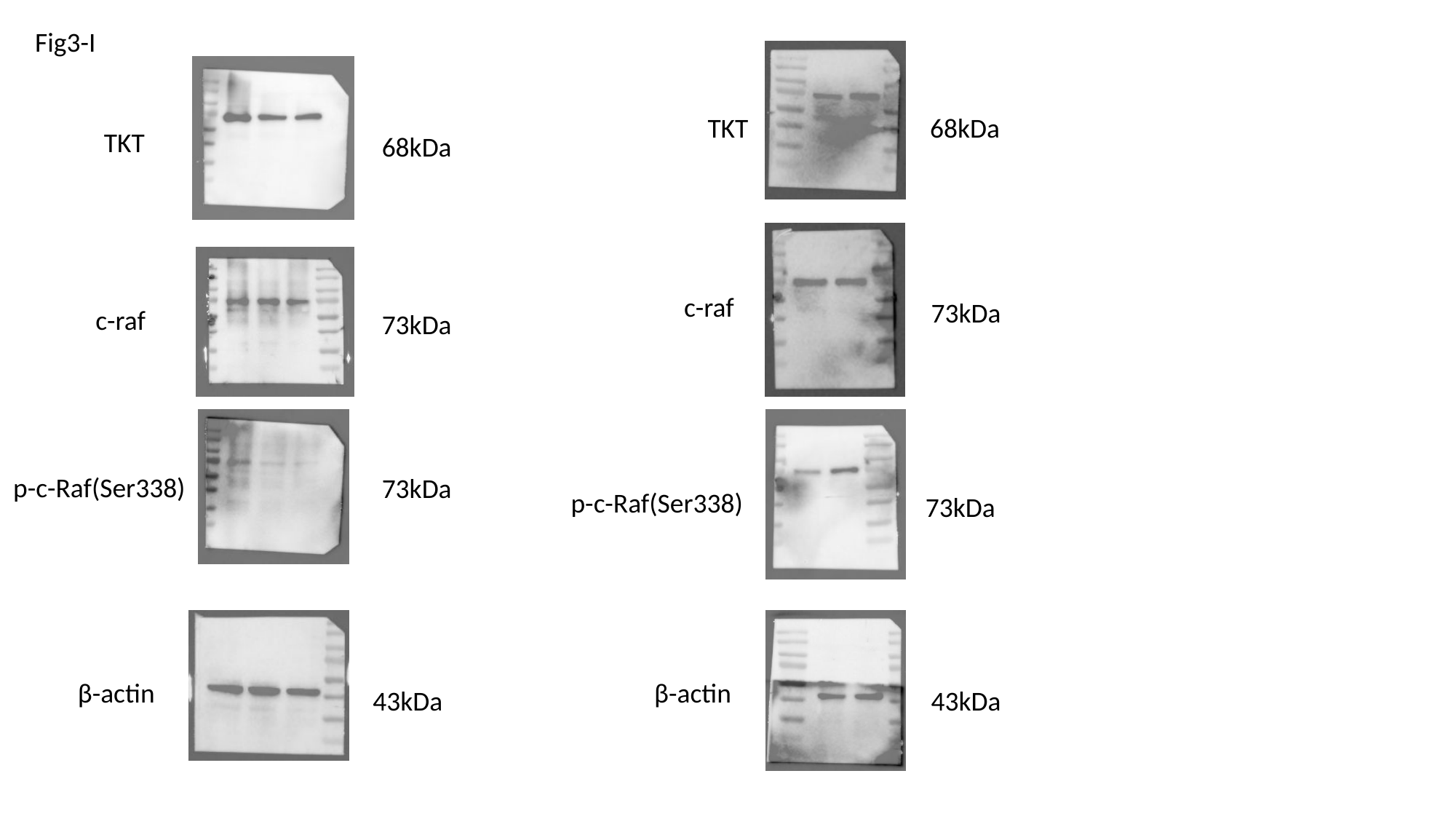

Fig3-I
TKT
68kDa
TKT
68kDa
c-raf
73kDa
c-raf
73kDa
p-c-Raf(Ser338)
73kDa
p-c-Raf(Ser338)
73kDa
β-actin
β-actin
43kDa
43kDa

## Slide 6
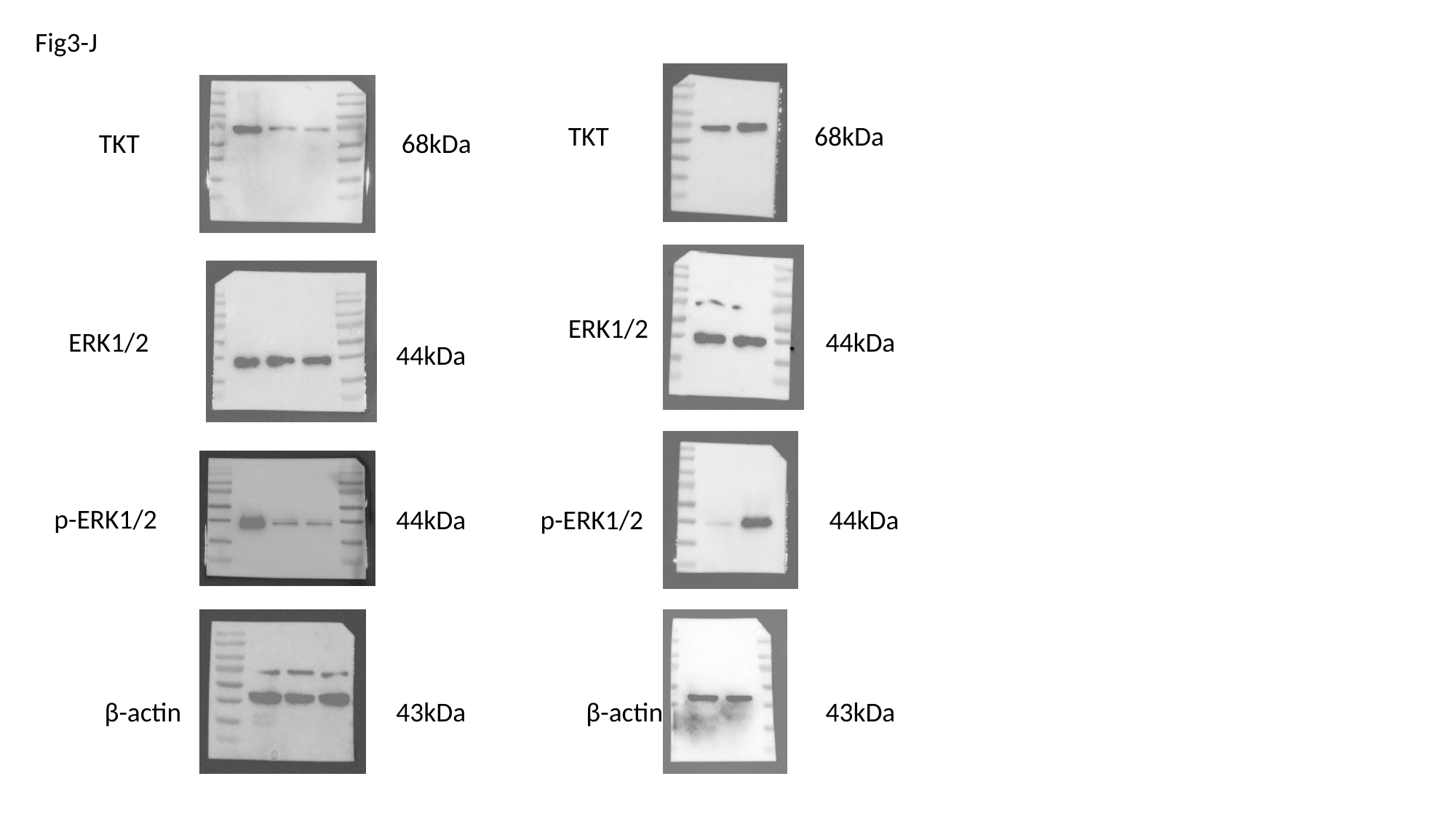

Fig3-J
TKT
68kDa
TKT
68kDa
ERK1/2
ERK1/2
44kDa
44kDa
p-ERK1/2
44kDa
p-ERK1/2
44kDa
β-actin
43kDa
β-actin
43kDa

## Slide 7
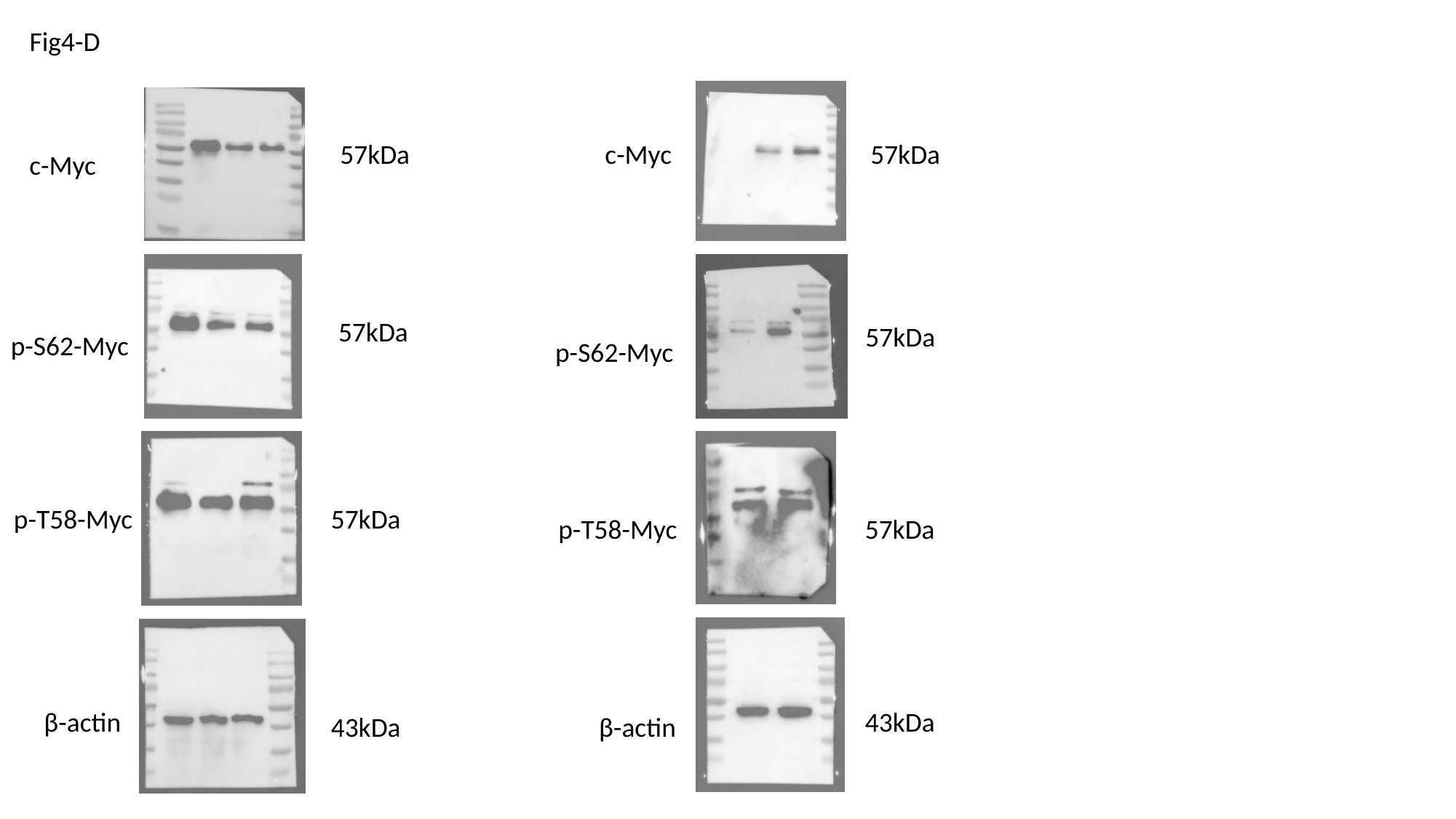

Fig4-D
57kDa
c-Myc
57kDa
c-Myc
57kDa
57kDa
p-S62-Myc
p-S62-Myc
p-T58-Myc
57kDa
p-T58-Myc
57kDa
β-actin
43kDa
43kDa
β-actin

## Slide 8
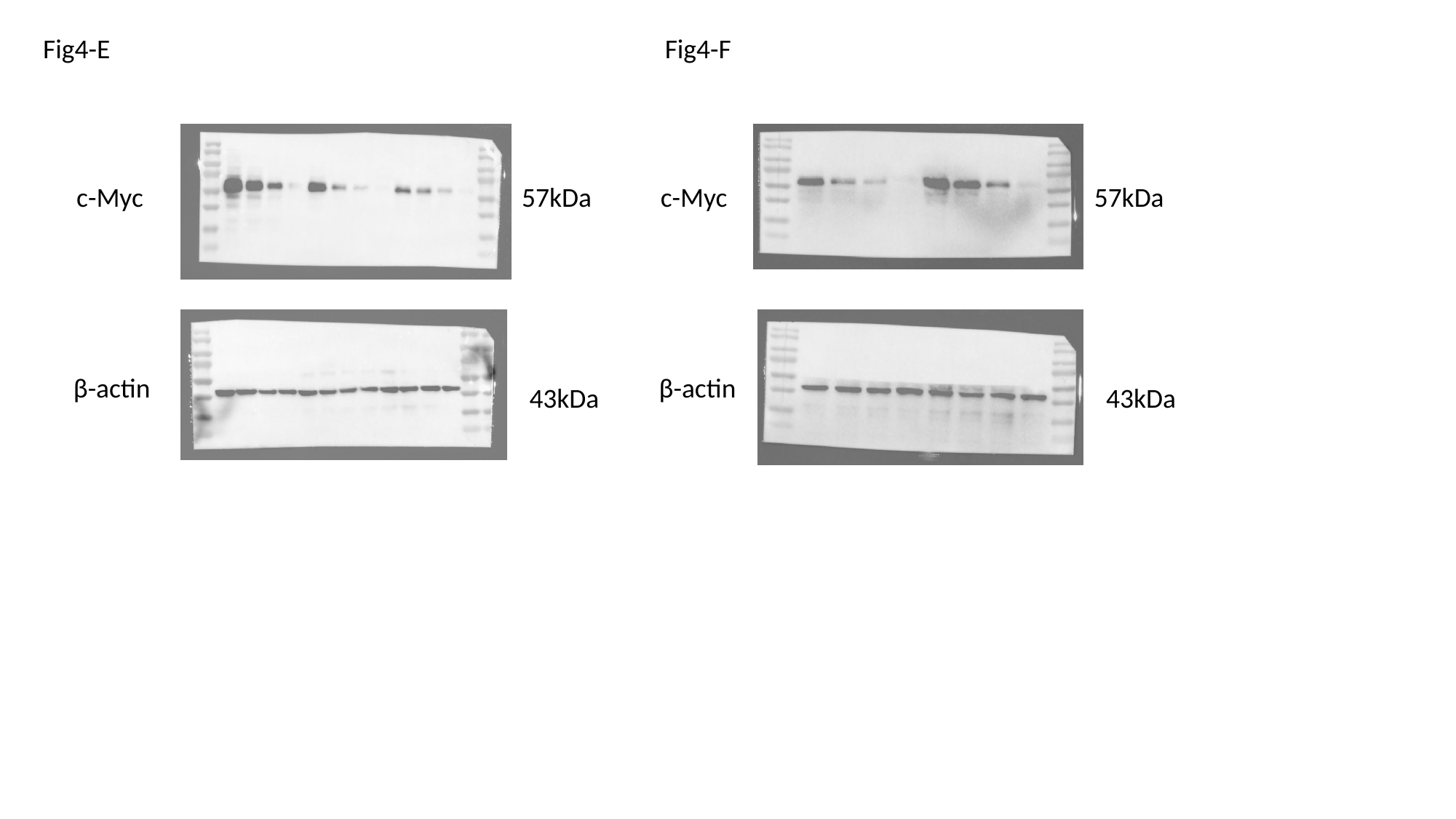

Fig4-E
Fig4-F
c-Myc
57kDa
c-Myc
57kDa
β-actin
β-actin
43kDa
43kDa

## Slide 9
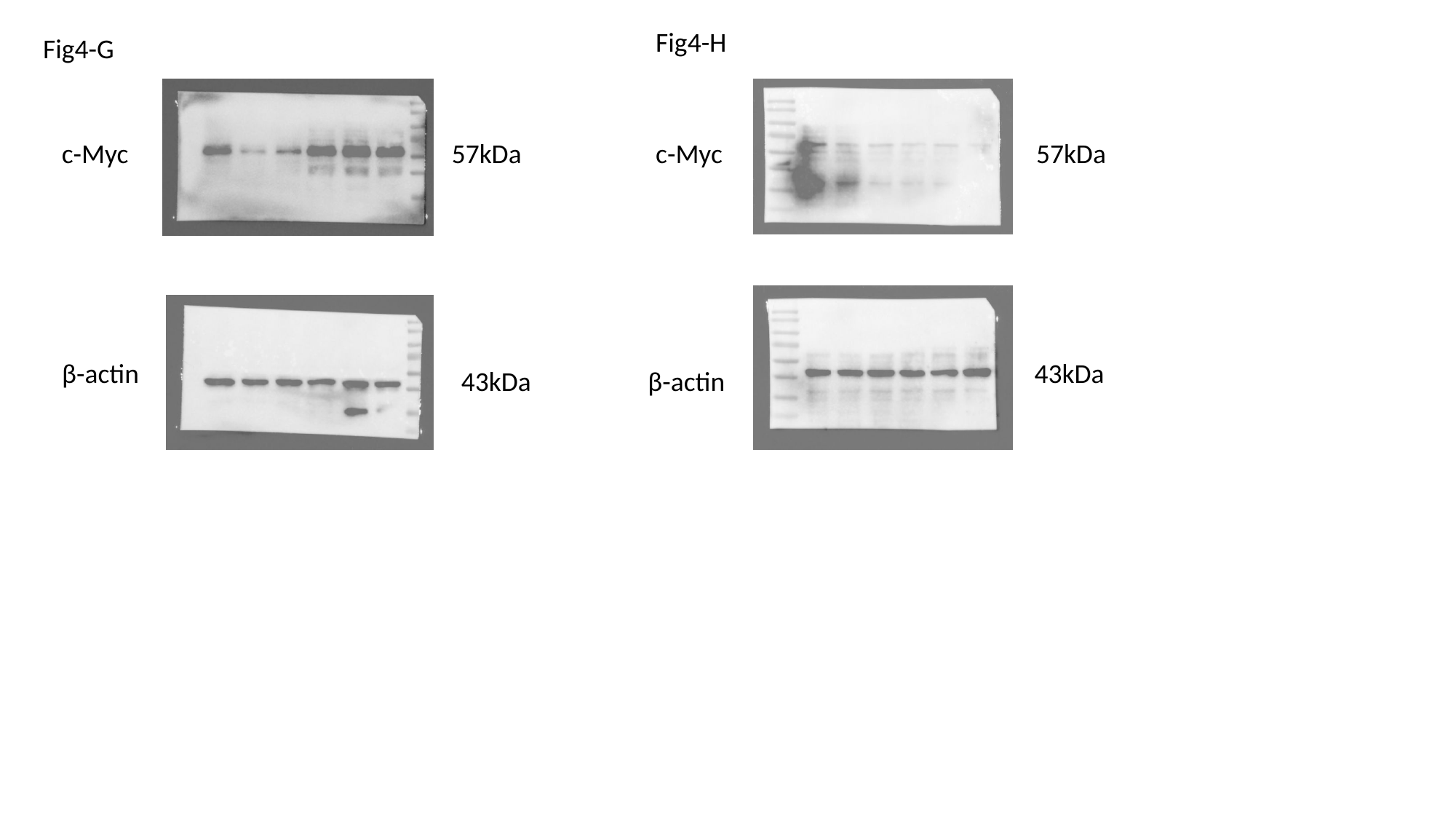

Fig4-H
Fig4-G
c-Myc
57kDa
c-Myc
57kDa
β-actin
43kDa
43kDa
β-actin

## Slide 10
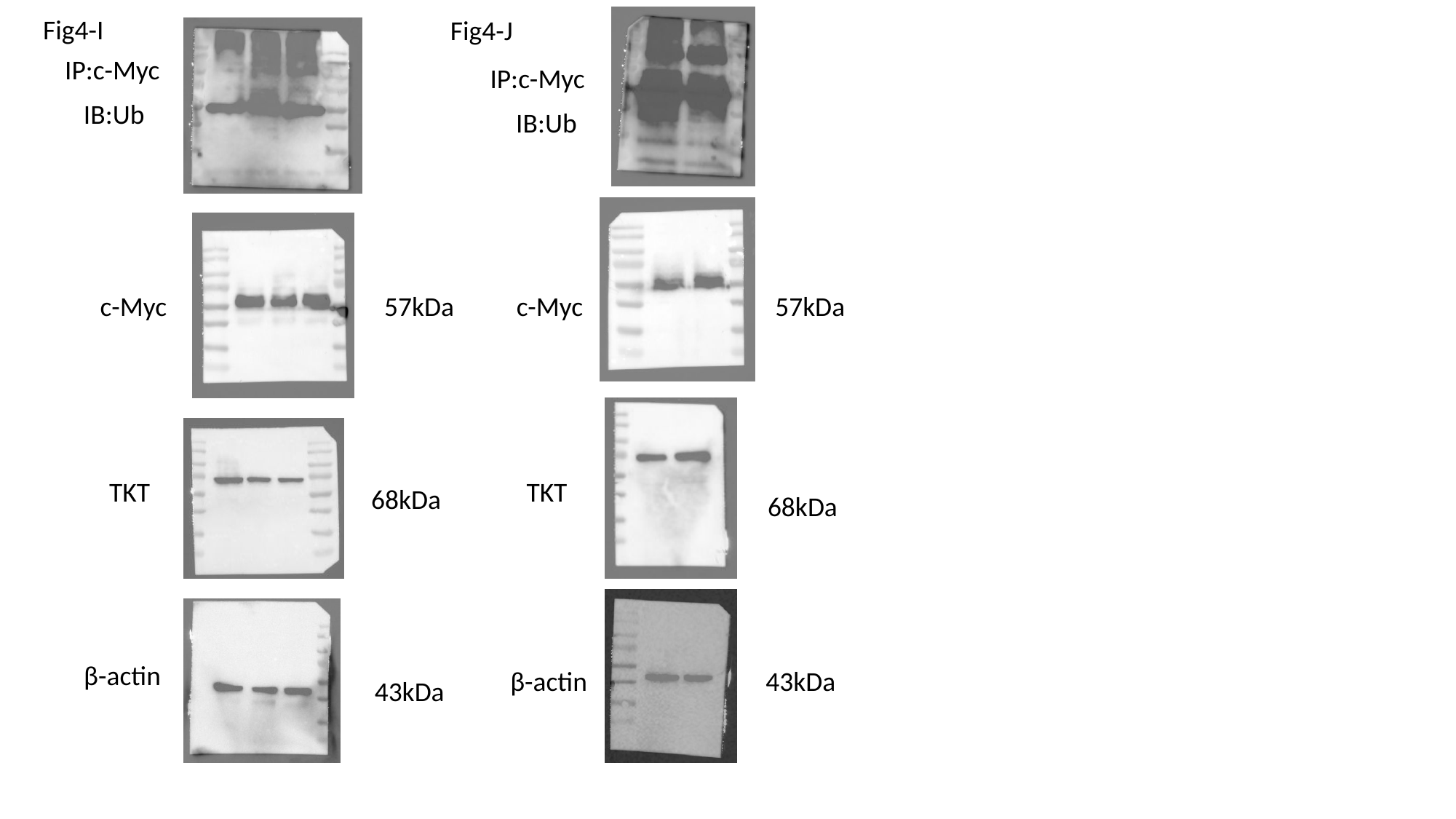

Fig4-I
Fig4-J
IP:c-Myc
IP:c-Myc
IB:Ub
IB:Ub
 c-Myc
57kDa
 c-Myc
57kDa
 TKT
 TKT
68kDa
68kDa
β-actin
β-actin
43kDa
43kDa

## Slide 11
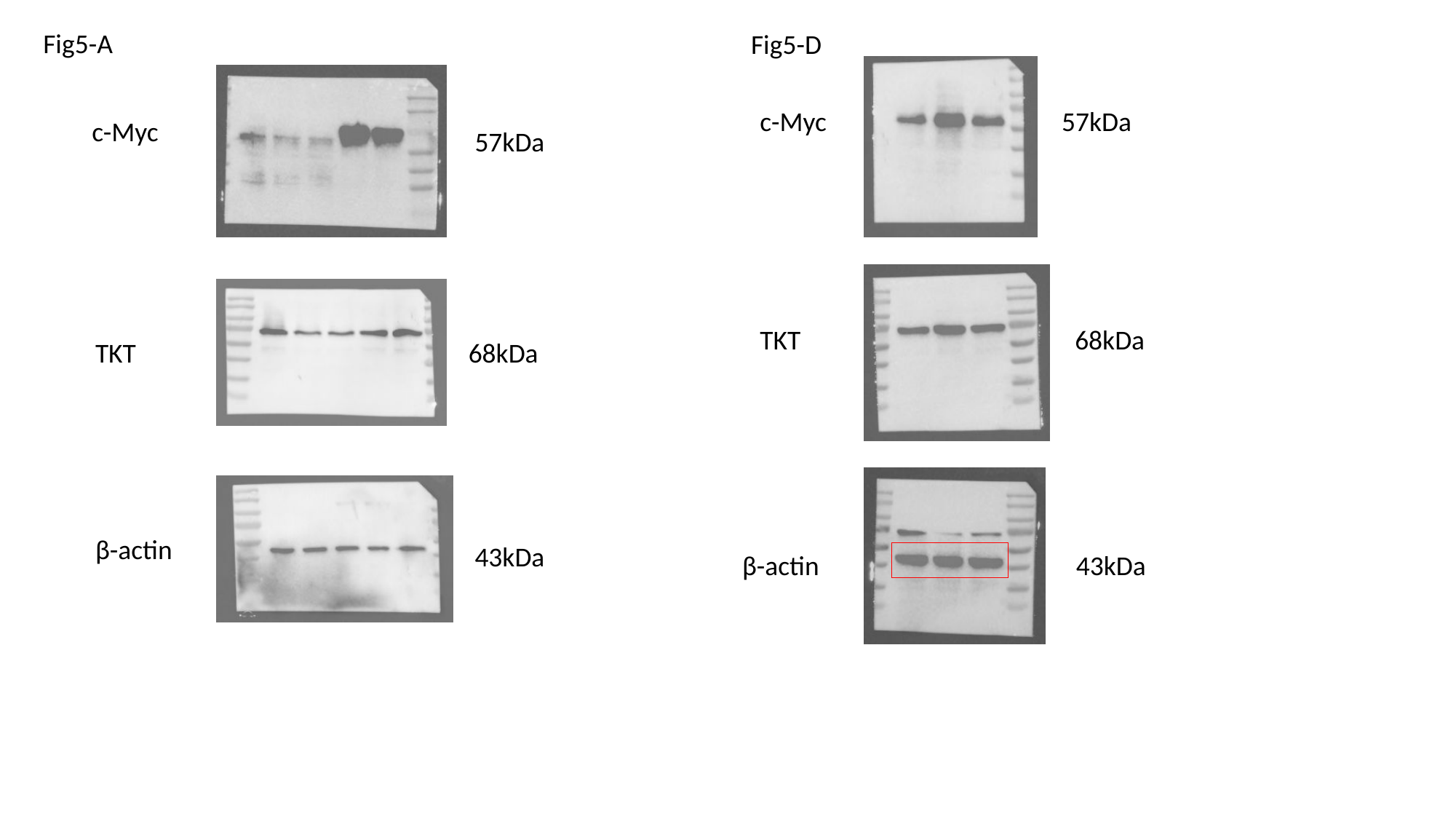

Fig5-A
Fig5-D
c-Myc
57kDa
c-Myc
57kDa
TKT
68kDa
TKT
68kDa
β-actin
43kDa
β-actin
43kDa

## Slide 12
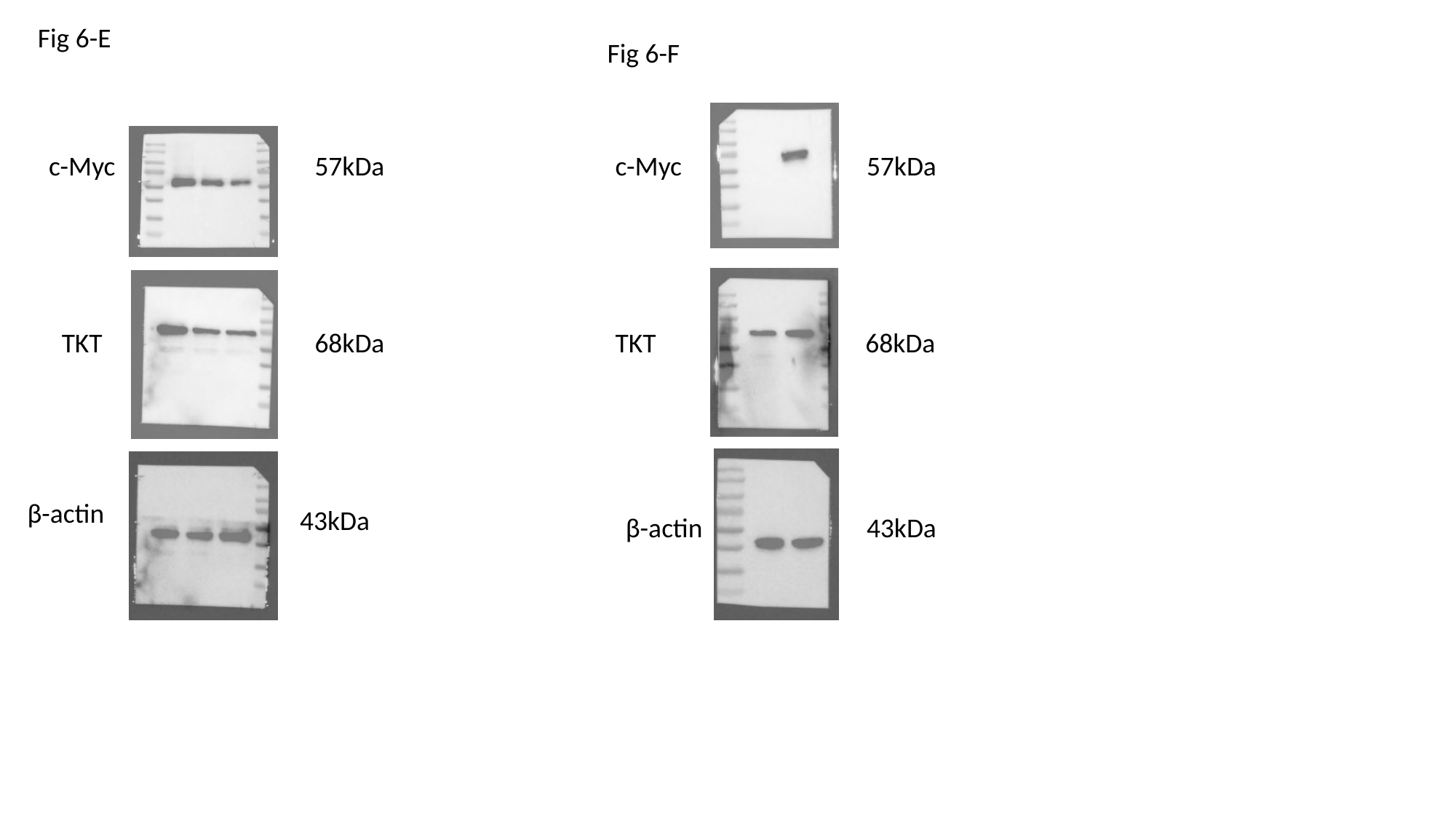

Fig 6-E
Fig 6-F
c-Myc
57kDa
c-Myc
57kDa
TKT
68kDa
TKT
68kDa
β-actin
43kDa
β-actin
43kDa
